# Oxygen evolution activity of nickel-based phosphates and effects of their electronic orbitals†

**DOI:** 10.1039/d4na00794h

**Published:** 2024-12-09

**Authors:** Yuuki Sugawara, Yuto Nakase, Gopinathan M Anilkumar, Keigo Kamata, Takeo Yamaguchi

**Affiliations:** a Laboratory for Chemistry and Life Science, Institute of Integrated Research, Institute of Science Tokyo Yokohama Kanagawa 226-8501 Japan sugawara.y.aa@m.titech.ac.jp yamag@res.titech.ac.jp; b R&D Centre, Noritake Co., Ltd Miyoshi Aichi 470-0293 Japan; c Laboratory for Materials and Structures, Institute of Integrated Research, Institute of Science Tokyo Yokohama Kanagawa 226-8501 Japan kamata.k.ac@m.titech.ac.jp

## Abstract

Metal phosphate-type compounds have been utilized in diverse applications, and their distinctive chemical properties have recently opened avenues for their use as catalysts. Metal phosphates have previously demonstrated significant electrocatalytic activity for the anodic oxygen evolution reaction (OER) in electrochemical water splitting. However, the critical factors influencing OER electrocatalysis on Ni-based phosphates have been insufficiently explored. We herein demonstrate nickel (Ni)-based phosphates—monoclinic Ni_3_(PO_4_)_2_, monoclinic Ni_2_P_2_O_7_, and monoclinic Ni_2_P_4_O_12_—as exemplary materials exhibiting outstanding OER activity in alkaline media. These Ni-based phosphates exhibit superior OER overpotentials compared to conventional Ni-based oxides (NiO) and phosphides (Ni_2_P). Additionally, their OER-specific activity surpasses that of the rare metal-based benchmark, IrO_2_, and previously reported state-of-the-art crystalline electrocatalysts comprising nonprecious metals. Long-term durability tests show that Ni_3_(PO_4_)_2_ maintains its OER activity even after 1000 repeated potential cycles while retaining its elemental composition and Raman spectrum. To understand the excellent OER activities of Ni-based phosphates, the atomic configurations within their crystals are examined. Remarkably, a clear correlation between Ni–O bond length and OER overpotentials is observed in both Ni-based phosphates and NiO, *i.e.*, shorter Ni–O bond lengths are highly beneficial for the OER. Density functional theory (DFT) calculations revealed that the outstanding OER activities of Ni-based phosphates are facilitated by their favorable electronic orbitals, which strengthen the Ni–O bond and improve the adsorption of OER intermediates on Ni sites. This mechanism is substantiated by DFT calculations employing surface slab models, where the adsorption of OER intermediates on the surface of Ni-based phosphates is more energetically favorable than on the surface of NiO. Hence, Ni-based phosphates are promising OER electrocatalysts, and this study provides important guidelines to further improve Ni-based electrocatalysts.

## Abstract 2

This study reports nickel-based phosphate nanoparticles as outstanding electrocatalysts for the oxygen evolution reaction and uncovers main factors determining their activities.

## Graphical abstract

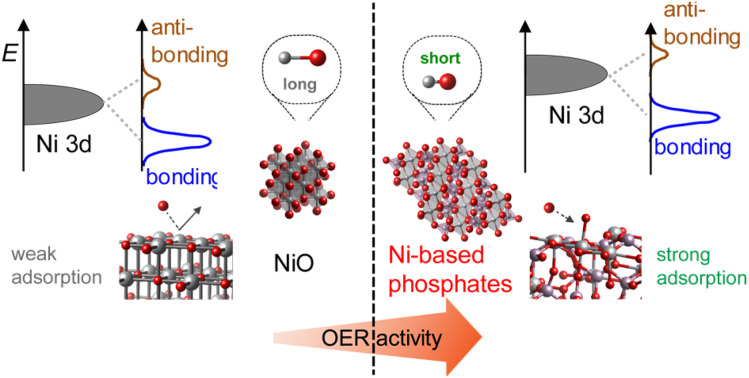

## Introduction

Solid-state metal phosphates, in which coordinated MO_*x*_ polyhedra (M = metal cations) and PO_4_ tetrahedra are connected *via* O atoms to form layered or 3D frameworks, have been used in various applications, such as dental implants,^1^ rechargeable metal-ion batteries,^2,3^ and supercapacitors.^4,5^ These compounds have many advantages, such as low cost, easy synthesis, thermal stability, unique redox properties, and a wide diversity of crystal structures and elemental compositions. Phosphate coordination can strengthen their crystal structures and stabilize the active metal oxidation states, leading to unique catalytic properties. Thus, metal phosphates have recently attracted considerable attention as catalysts^6–11^ because present energy issues have increasingly stimulated the demand for the development of clean and sustainable energy platforms that can achieve net zero carbon emissions, which is the most urgent global mission. One of the examples of the application of metal phosphates to catalytic energy conversion reactions is their use as electrocatalysts for electrochemical hydrogen production. Utilizing hydrogen as an energy carrier is a highly promising option for the construction of a sustainable circular carbon economy, given its status as a carbon-free chemical fuel boasting a high weight energy density (142 MJ kg^−1^).^12^ However, hydrogen is mainly produced by reforming fossil fuels at present, exacerbating global warming and energy resource depletion. Therefore, the development of a sustainable and cost-effective technology for large-scale hydrogen production is essential.

An exemplary approach to green hydrogen production involves the electrolysis of earth-abundant water using renewable energy-derived electricity. Anion-exchange membrane water electrolyzers (AEMWEs) operate at low temperatures (<100 °C) in alkaline environments and can employ inexpensive nonprecious metals, rendering them a large-scale and cost-effective device.^13^ AEMWEs use anion-exchange membranes (AEMs) to separate electrodes, limiting gas crossover and enabling operation at high pressure.^14^ Recent advancements have yielded highly OH^−^-conductive and durable AEMs for the AEMWE systems.^15–18^ However, achieving high electrolysis performances without using precious metal-based electrocatalysts, such as platinum, iridium, and ruthenium-based compounds, remains challenging. The anodic four-electron transfer oxygen evolution reaction (OER: 4OH^−^ → O_2_ + 2H_2_O + 4e^−^) in water splitting has a larger overpotential compared to the cathodic two-electron transfer hydrogen evolution reaction, presenting a significant bottleneck for the efficiency of AEMWEs. Thus, highly active and inexpensive electrocatalysts for the OER are desired to address this concern.

As OER electrocatalysts, 3d transition metal-based compounds have recently drawn much interest owing to their earth-abundance and cost-effectiveness. These OER electrocatalysts include various types, such as oxides,^19–21^ hydroxides,^22–25^ sulfides,^26,27^ nitrides,^28,29^ phosphides,^30–32^ and chalcogenides.^33^ Recently, our group has intensively studied cost-effective iron (Fe)-based oxides for OER electrocatalysis, and we have reported highly active Fe-based multimetal oxides using in-house-proposed structure-based descriptors.^34,35^ Meanwhile, metal phosphates have also been employed as OER electrocatalysts. For instance, NaCo(PO_3_)_3_ (ref. 36) and Na_2_CoP_2_O_7_ (ref. 37) have emerged as highly active OER electrocatalysts owing to the specific Co–O coordination environments of CoO_6_ and CoO_5_ polyhedra, respectively. Similarly, Co_3_(PO_4_)_2_ showcased prominent OER activity attributed to the presence of CoO_5_ hexahedra.^38^ Among 3d transition metals, nickel (Ni) has drawn much interest as an active metal for the OER process because Ni intrinsically catalyzes the OER with higher efficiency compared with other nonprecious transition metals.^39^ There exit many reports on the OER activity of a series of Ni-based perovskite oxides^19,40,41^ and oxyhydroxides,^22,42,43^ such as LaNiO_3_ and NiOOH, respectively, with their activity trends explained by structural and/or electronical factors. Incorporating or substituting metallic components in such metal oxides can modify their electronic states, thus improving OER activity. This approach has also been introduced for designing Ni-based phosphates as OER electrocatalysts, such as Ni_*x*_Co_3−*x*_(PO_4_)_2_ (ref. 44) and Ni_3_(PO_4_)_2_(Na)(Co_*x*_Fe_*x*_).^45^ However, the important factors influencing OER electrocatalysis on Ni-based phosphates have been scarcely investigated.

**Fig. 1:**
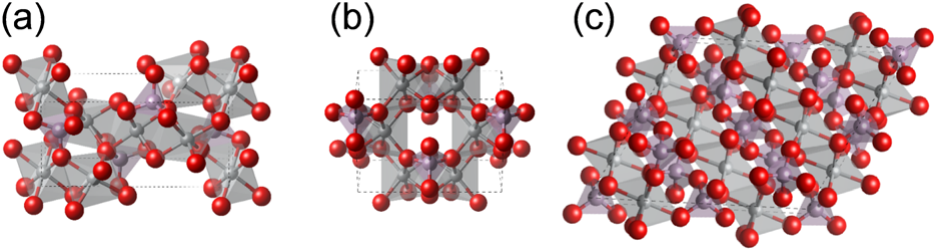
Crystal structures of Ni-based phosphates. (a) Ni_3_(PO_4_)_2_, (b) Ni_2_P_2_O_7_, and (c) Ni_2_P_4_O_12_. The gray, purple, and red spheres represent Ni, P, and O atoms, respectively.

Herein, we present a detailed investigation into OER electrocatalysis on Ni-based phosphates, elucidating the main factors influencing their OER performance. We focus on three types of Ni-based phosphates, as shown in Fig. 1: monoclinic Ni_3_(PO_4_)_2_, monoclinic Ni_2_P_2_O_7_, and monoclinic Ni_2_P_4_O_12_. Because these Ni-based phosphates comprise only Ni as the metallic component, the effect of other elements on the catalytic performance can be eliminated, which enables us to clearly elucidate structural and electronical factors for OER electrocatalysis. We synthesize these Ni-based phosphates and evaluate their OER performances in alkaline media. Furthermore, we evaluate their durability through potential cycles and chronoamperometry under different potentials. Finally, to understand the excellent OER performances of Ni-based phosphates, we conducted density functional theory (DFT) calculations and discuss the main factors determining their OER activities in terms of their crystal structures and electronic orbitals.

## Materials and methods

### Chemicals

Ni(OAc)_2_·4H_2_O (98%), NH_4_H_2_PO_4_ (99%), and d,l-malic acid (MA) (99%) were purchased from Kanto Chemical Co., Inc. (Tokyo, Japan). NiO (product ID: 637130, *S*_BET_ = 82 m^2^ g^−1^), Nafion_aq_ (product ID: 274704), and gold nanopowder (Au, product ID: 636347) were purchased from Sigma-Aldrich, Inc. (St. Louis, MO, USA). The phase purity of NiO and metallic Au was confirmed *via* X-ray diffraction (XRD), as shown in Fig. S1 and S2.† Carbon nanotubes (CNTs, 90%) were purchased from Nanocyl SA (Sambreville, Belgium) and functionalized as previously described.^46^ KOH (85%) and all other chemicals were purchased from FUJIFILM Wako Pure Chemical Corporation (Osaka, Japan) and used as received. Ni_2_P (*S*_BET_ = 11 m^2^ g^−1^) was synthesized according to literature protocols.^47^ The phase purity of Ni_2_P was confirmed *via* XRD, as shown in Fig. S3.†

### Instruments

XRD analysis, Brunauer–Emmett–Teller (BET) surface area (*S*_BET_) measurements, scanning electron microscopy (SEM), transmission electron microscopy (TEM), high-angle annular dark-field scanning TEM (HAADF-STEM) measurements, energy-dispersive X-ray (EDX) spectroscopy, inductively coupled plasma-atomic emission spectroscopy (ICP-AES), and Raman spectroscopy were performed using the instruments described in previous studies.^35,48^ Detailed methodologies are presented in the ESI.†

### Synthesis

Ni_3_(PO_4_)_2_, Ni_2_P_2_O_7_, and Ni_2_P_4_O_12_ were synthesized through the MA-aided sol–gel method with modifications based on our previous study.^49^ Ni(OAc)_2_·4H_2_O (10 mmol), NH_4_H_2_PO_4_, and MA were dissolved in 50 mL of deionized water. The molar ratios of Ni/P/MA were adjusted according to Table 1. Subsequently, the solutions were evaporated at 80 °C to obtain dried solids, followed by calcination in an ambient atmosphere at the specified temperature for 5 h, as described in Table 1. This process yielded Ni_3_(PO_4_)_2_, Ni_2_P_2_O_7_, and Ni_2_P_4_O_12_ as yellow, yellow, and gray powders, respectively.

**Table 1:** Conditions for the synthesis of Ni-based phosphates

Catalyst	Feed molar ratio [—]			Calcination temp. [°C]
	Ni	P	MA	
Ni_3_(PO_4_)_2_	6	4	15	800
Ni_2_P_2_O_7_	1	1	3	700
Ni_2_P_4_O_12_	2	4	9	800

### Electrochemical measurements

The OER activities of the synthesized catalysts were measured using a HZ-7000 (MEIDEN HOKUTO CORPORATION, Tokyo, Japan) and a rotating-disk electrode (RDE, HR2-D1-GC5, disk area: 0.196 cm^2^) equipped with an electrode rotator (HR-500). The measurement setup is depicted in Fig. S4.† Hg/HgO (1 M KOH) and Pt wire were used as the reference and counter electrodes, respectively. The electrolytic cell featured a gas inlet to saturate the cell *via* N_2_ bubbling. The Hg/HgO electrode was calibrated, following previously described methods^48^ to express the reversible hydrogen electrode (RHE) potential using eqn (1).

1 *E*_RHE_ = *E*_Hg/HgO_ + 0.9047 V

The preparation of catalyst layers on the RDE as the working electrode was performed as follows. 0.25 mg of catalyst, 2.25 mg of functionalized CNTs, 0.144 mL of K^+^-exchanged Nafion_aq_ (3.3 wt%), 1.2 mL of EtOH, and 1.2 mL of pure water were mixed, homogenized for 3 min in an ice bath and then ultrasonicated for 2 h to obtain catalyst inks. Subsequently, 10 μL of the ink was coated onto the RDE and dried. RDEs for durability tests were prepared using Au as a conductive support instead of CNTs as CNTs can undergo corrosion at high potentials during prolonged OER measurements.^50^ A mixture of 2 mg of catalyst powder, 8 mg of Au, 300 μL of 3.3 wt% K^+^-ion exchanged Nafion_aq_, 0.68 mL of isopropanol and 1.32 mL of water was homogenized and ultrasonicated to prepare the catalyst ink, followed by the same protocol as described above. The RDEs were prescanned *via* five cyclic voltammetry (CV) sweeps between 0.1 and 1.2 V *vs.* RHE at a sweep rate of 50 mV s^−1^ in 1 M KOH. The initial OER catalytic activity was evaluated using CV at 10 mV s^−1^ between 1.2 and 1.8 V *vs.* RHE with a rotation speed of 1600 rpm in 1 M KOH at room temperature. Polarization curves for the OER were obtained by averaging the forward and backward sweeps of the 10th CV cycle to eliminate capacitive currents from the measured electrodes. *iR* drops were compensated using the following eqn (2):

2 *iR*-compensated *E* = *E*_RHE_ − *iR*

where *i* and *R* are the recorded current and solution resistance measured using AC impedance, respectively. To compare the intrinsic OER activities, the measured *i* was normalized by the *S*_BET_ of each catalyst, allowing for the calculation of the OER-specific activities. After 10 CV cycles, the catalyst was retrieved from the RDE and analyzed by STEM-EDX and Raman spectroscopy to identify actual active species involved in OER electrocatalysis. Catalyst durability was assessed by performing 1000 repeated potential cycles at 100 mV s^−1^ between 1.2 and 1.7 V *vs.* RHE in 1 M KOH, as well as chronoamperometry at 2 V in 1 M KOH and at 1.9 V in 0.01 M K_2_CO_3_.

### Thermodynamic study

A Pourbaix diagram for the Ni–P–H_2_O system was simulated using HSC Chemistry 10.4.1.1 version software^51^ (Metso Corporation, Espoo, Finland) to study the theoretical stability of Ni phosphates under OER conditions in alkaline media, *i.e.*, in high-pH and high-potential regions. The simulations were conducted at 298.15 K, and the diagram was referenced to the standard hydrogen electrode (SHE).

### DFT calculations

DFT calculations were executed using the Vienna *Ab initio* Simulation Package (VASP 6.1.0).^52–55^ The details of these calculations are presented in the ESI.† The unit cells and atomic positions of the Ni-based phosphates and NiO were optimized. Initial structures of the compounds were collected from the Inorganic Crystal Structure Database (ICSD): Ni_3_(PO_4_)_2_ (ICSD: 153159), Ni_2_P_2_O_7_ (ICSD: 30433), Ni_2_P_4_O_12_ (ICSD: 409092), and NiO (ICSD: 28834). The structures and dimensions of the unit cells are depicted in Fig. S5a† and summarized in Table S1.† After geometrical optimization, the occupied Ni 3d band centers were extracted from the projected density of states (PDOS).^56^

For surface calculations, the aforementioned geometrically optimized bulk structures were cleaved along the (10 −1) and (100) facets for Ni_2_P_4_O_12_ and NiO, respectively, to construct their surface slab models. The slabs were separated by a 15 Å vacuum layer. The surface slab models are depicted in Fig. S5b.† The adsorption energy of the O* intermediate (Δ*E*_O*_), was calculated using eqn (3):^57^

3 Δ*E*_O*_ = *E*(O*) − *E*(*) − (*E*_H_2_O_ − *E*_H_2__)

where *E*(O*) and *E*(*) represent the total energies of the surface with adsorbed O* intermediates and the pristine surface, respectively. *E*_H_2_O_ and *E*_H_2__ denote the total energies of gaseous H_2_O and H_2_ molecules, respectively.

## Results and discussion

### Characterization of synthesized catalysts

**Fig. 2:**
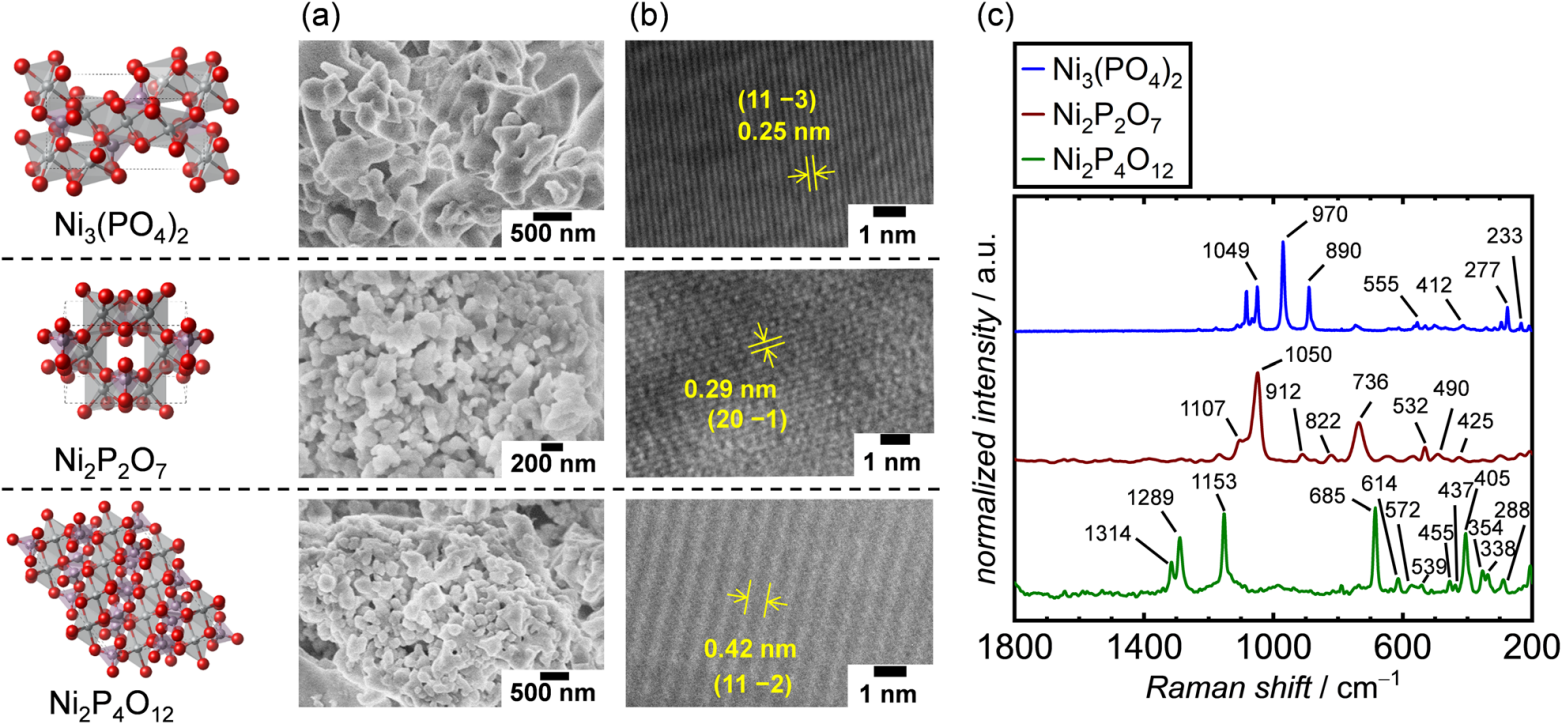
Characterization results of Ni-based phosphates. (a) SEM and (b) TEM images and (c) Raman spectra of the Ni-based phosphates.

The crystal structures of the synthesized Ni-based phosphates were characterized using XRD measurements. Fig. S6–S8† show the XRD patterns of the Ni-based phosphates, indexed to the monoclinic Ni_3_(PO_4_)_2_ (ICSD: 153159, space group: *P*2_1_/*c*), monoclinic Ni_2_P_2_O_7_ (ICSD: 30433, space group: *C*2/*m*), and monoclinic Ni_2_P_4_O_12_ (ICSD: 409092 space group: *C*2/*c*), respectively. The crystallite diameter (*d*_XRD_) for the Ni-based phosphates was calculated using Scherrer's equation. SEM images of the Ni-based phosphates, depicted in Fig. 2a, reveal aggregated spherical particles with sizes ranging from 70–400 nm. Furthermore, their TEM images shown in Fig. 2b display lattice fringes with *d*-spacing values of 0.25, 0.29, and 0.42 nm, corresponding to (11 −3), (20 −1), and (11 −2) facets for Ni_3_(PO_4_)_2_, Ni_2_P_2_O_7_, and Ni_2_P_4_O_12_, respectively, consistent with the XRD data. Additionally, the Ni-based phosphates possessed theoretical Ni : P compositions, according to ICP-AES results. Moreover, their measured *S*_BET_ values ranged from 2–10 m^2^ g^−1^, corresponding to their particle sizes. The *S*_BET_ of the catalysts was exploited to normalize their OER current density for the comparison of their surface area-specific activities for the OER in the following sections. Table 2 summarizes the aforementioned characterization of the three types of Ni-based phosphates. Additionally, Raman spectra of the Ni-based phosphates (Fig. 2c) were consistent with the reported characteristic Raman shifts for these compounds, as shown in Table S2.† The XRD patterns and Raman spectra confirmed the successful syntheses of Ni_3_(PO_4_)_2_, Ni_2_P_2_O_7_, and Ni_2_P_4_O_12_.

**Table 2:** Characterization results for the Ni-based phosphates

Catalyst	Structure	*d*_XRD_ [nm]	*d*_SEM_ [nm]	Elemental composition (ICP) [wt%]	*S*_BET_ [m^2^ g^−1^]
Ni_3_(PO_4_)_2_	Monoclinic	81 (100)	200–400	Theor.: Ni 74.0, P 26.0	2
				Exptl.: Ni 73.6, P 26.4	
Ni_2_P_2_O_7_	Monoclinic	30 (110)	70–200	Theor.: Ni 65.5, P 34.5	10
				Exptl.: Ni 66.8, P 33.2	
Ni_2_P_4_O_12_	Monoclinic	67 (11 −1)	100–300	Theor.: Ni 48.7, P 51.3	4
				Exptl.: Ni 41.3, P 58.7	

### OER activities

**Fig. 3:**
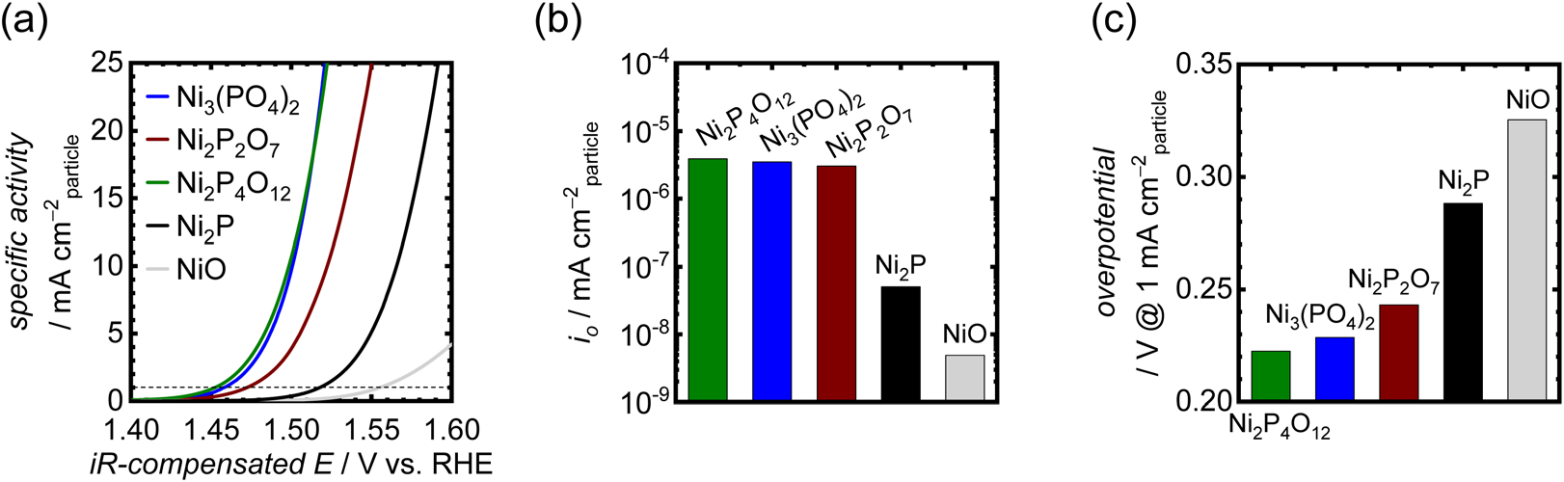
(a) Polarization curves showing OER-specific activities for the Ni-based phosphates, NiO, and Ni_2_P in 1 M KOH. (b) *i*_o_ values for the OER of the Ni-based phosphates, NiO, and Ni_2_P. (c) Comparison of their OER overpotentials at 1 mA cm_particle_^−2^.

The OER activities of the synthesized Ni-based phosphates were measured using RDEs in 1 M KOH. In the OER measurements, catalyst particles were mixed with CNTs as a conductive support to fabricate electrodes, as the pristine Ni-based phosphate possessed inadequate electrical conductivity and exhibited much lower current density without CNTs as shown in Fig. S9,† thereby necessitating their combination with CNTs. Furthermore, unmixed CNTs displayed minimal current due to carbon oxidation, compared to the mixed sample, as depicted in Fig. S10.† This result indicates that CNTs do not possess catalytic activity and solely play the role of an electrically conductive material. The mixing ratio of catalyst–CNT was optimized to 1 : 9, resulting in the maximum current output. Fig. 3a compares OER activities of the three Ni-based phosphates, NiO and Ni_2_P. To eliminate the effects of the surface area of the catalyst particles on OER activity, the recorded OER currents were normalized using the *S*_BET_ of the catalyst particles. The electrochemically active surface area (ECSA) is generally preferred over *S*_BET_ for calculating specific activity because not all N_2_ adsorption sites are electrochemically active.^58^ However, our electrode samples were mixed with a large number of CNTs, which possess a much higher surface area. Therefore, the measured ECSAs of the electrode by the double layer capacitance method include those of CNTs, making it challenging to separate them from the ECSAs of phosphate catalysts. Hence, we opted to use the *S*_BET_ of phosphate catalysts to normalize their OER activities. Consequently, the *iR*-compensated polarization curves based on the OER-specific activity are shown in Fig. 3a, derived from the average of the 10th forward and backward CV scans. Ni_3_(PO_4_)_2_ and Ni_2_P_4_O_12_ exhibited very low OER onset potentials of around 1.43 V *vs.* RHE. Notably, the OER activity of the Ni-based phosphates was much better than those of NiO and Ni_2_P. NiO and Ni_2_P demonstrated oxidation peaks for the Ni(ii)/Ni(iii) redox couple in the range of 1.35–1.40 V, as displayed in Fig. S11,† whereas the Ni-based phosphates displayed negligible peaks, implying distinct redox characteristics for the Ni-based phosphates. In addition, complementary Tafel plots for the samples were used to obtain the exchange current density (*i*_o_), as shown in Fig. S12.†*i*_o_ values for the Ni-based phosphates were much higher than those for NiO and Ni_2_P, as shown in Fig. 3b, indicating the superior OER kinetics of Ni-based phosphates. Their OER overpotentials at 1 mA cm_particle_^−2^ are displayed in Fig. 3c. Notably, the order of OER activities among the Ni-based phosphates did not correlate with the Ni : P ratios, suggesting that this trend cannot be solely explained using compositional effects.

To identify the real active species on the Ni-based phosphate electrodes for OER electrocatalysis, STEM-EDX measurements were performed using the catalysts recovered from the RDEs after 10 CV cycles of OER measurements. Fig. S13a, c, and e† depict the EDX elemental mappings of Ni_3_(PO_4_)_2_, Ni_2_P_2_O_7_, and Ni_2_P_4_O_12_ after 10 cycles under high potential in alkaline media, revealing that the catalyst particles maintained similar compositions of Ni and P to those of the initial particles. In addition, Raman measurements conducted on the catalysts before and after 10 cycles of OER measurements indicated that the initial phases of Ni_3_(PO_4_)_2_, Ni_2_P_2_O_7_, and Ni_2_P_4_O_12_ remained intact after OER measurements, as shown in Fig. S13b, d, and f.† Previous studies have reported thorough dissolution of P from Co- and Fe-based phosphates during OER measurements, resulting in the electrochemical generation of Co- and Fe-based (oxyhydr)oxides.^48,59,60^ Contrarily, the aforementioned results indicate negligible leaching of P from both the bulk and surface of the particles during the OER in the case of Ni-based phosphates. A previous study reported partial P leaching from the particle surface of an “amorphous” Ni-based phosphate (Ni_3_P_2_O_*x*_),^61^ whereas the Ni-based phosphates in this study did not exhibit notable P leaching even from the surface owing to their stable crystalline structures.

**Fig. 4:**
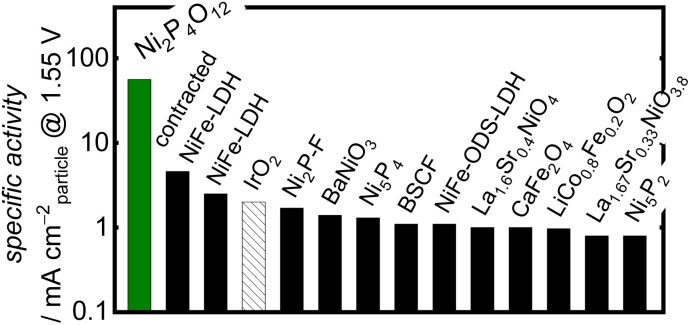
Comparison of the OER-specific activity of Ni_2_P_4_O_12_ in alkaline media with the state-of-the-art crystalline nonprecious metal-based catalysts, BSCF, and IrO_2_. Further details of the cited literature are presented in the ESI.†

When comparing the Ni-based phosphates with the previously reported benchmark nonprecious multimetal oxide, BSCF, the benchmark precious metal oxide, IrO_2_, and state-of-the-art OER electrocatalysts comprising nonprecious metals, the OER-specific activity of the Ni-based phosphates was found to be 10^1^–10^2^ fold higher than those of other OER electrocatalysts, as shown in Fig. 4 and Table S3.† To the best of our knowledge, the OER-specific activity of Ni_2_P_4_O_12_ surpassed that of any previously reported crystal particle-type OER electrocatalysts. Hence, this study demonstrates that Ni-based phosphates are promising compounds with intrinsic activity as OER electrocatalysts.

### Stability of Ni-based phosphates during the OER

**Fig. 5:**
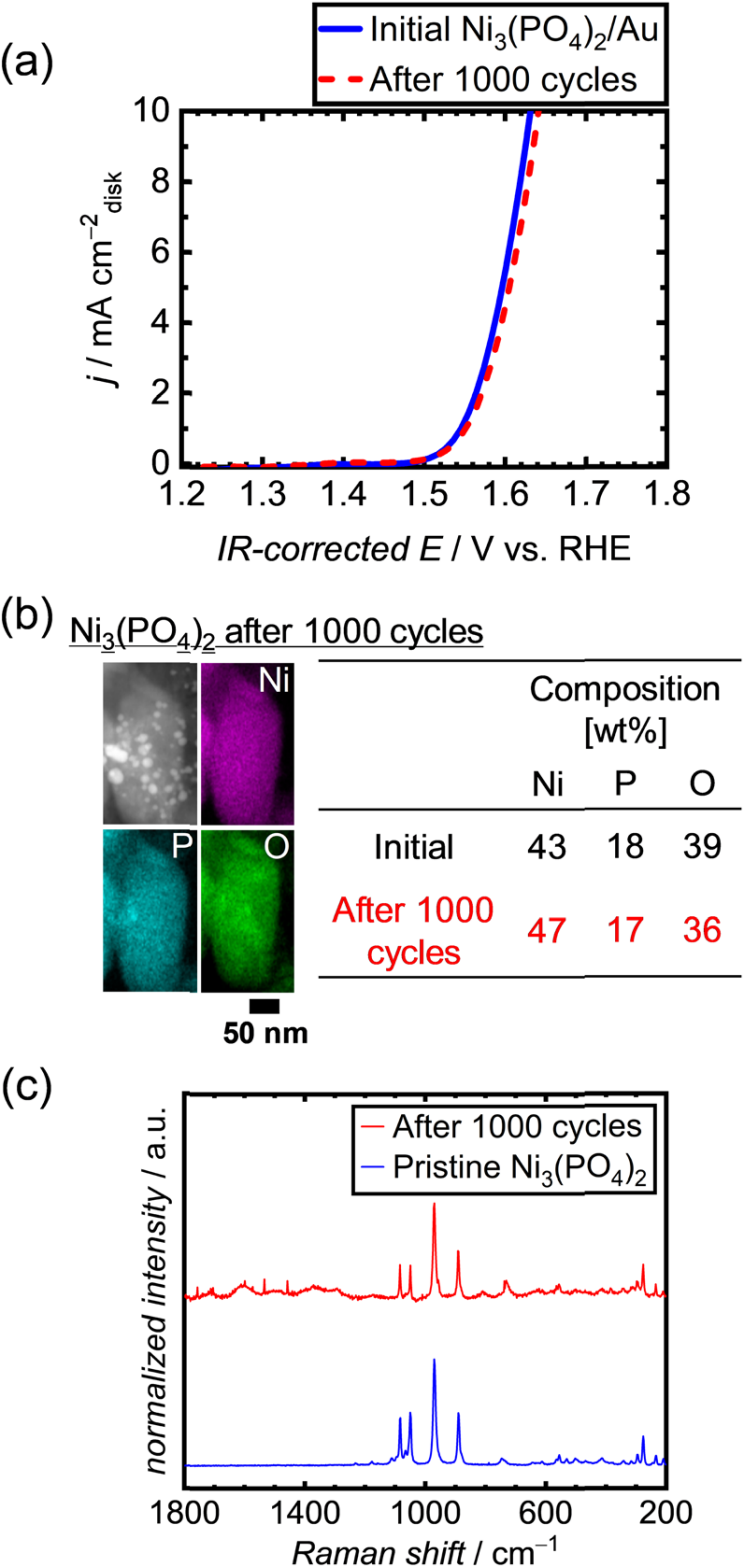
(a) Polarization curves of Ni_3_(PO_4_)_2_/Au after 1000 repeated potential cycles between 1.2 and 1.7 V *vs.* RHE in 1 M KOH. (b) STEM-EDX image and (c) Raman spectrum of Ni_3_(PO_4_)_2_/Au after 1000 potential cycles.

To examine the long-term durability of the Ni-based phosphates under OER conditions, an alternative conductive support was used instead of CNTs. This decision was made owing to the corrosive nature of the carbon support under OER potential, which can lead to the deterioration of electrocatalytic performances.^50^ Therefore, a highly corrosion-resistant conductive support was needed to test the durability of the OER electrocatalyst, replacing CNTs. We thus employed metallic Au, which has excellent electrical conductivity and good corrosion/dissolution resistance^62^ under OER potentials up to 2 V in 1 M KOH.^63^ In addition, Au is an intrinsically OER-inactive metal,^64,65^ making it suitable for the examination of electrocatalyst performances. We employed a commercial Au nanopowder with a particle size of 30–50 nm, as depicted in Fig. S14a.† Among the three types of Ni-based phosphates, we selected Ni_3_(PO_4_)_2_ for the long-term tests because of its best theoretical stability under OER conditions. The initial OER activity of the Ni_3_(PO_4_)_2_/Au electrode without CNTs is displayed in Fig. S14b,† which is much higher than that of the Au electrode without Ni_3_(PO_4_)_2_, indicating that Au does not intrinsically catalyze the OER compared to Ni_3_(PO_4_)_2_, but rather serves as a conductive support. The OER polarization curves of Ni_3_(PO_4_)_2_/Au after repeated 1000 potential cycles, shown in Fig. 5a, demonstrate that the OER performance of Ni_3_(PO_4_)_2_ did not deteriorate over time. Fig. 5b exhibits STEM-EDX images of Ni_3_(PO_4_)_2_/Au after 1000 cycles, indicating that the elemental compositions of Ni and P in Ni_3_(PO_4_)_2_ remained consistent during the repeated potential cycles in the OER region, *i.e.*, 1.2–1.7 V *vs.* RHE. Furthermore, the Raman spectrum of Ni_3_(PO_4_)_2_/Au after 1000 cycles exhibits characteristic peaks identical to those of pristine Ni_3_(PO_4_)_2_, as shown in Fig. 5c. These results demonstrate the long-term stability of Ni_3_(PO_4_)_2_ for OER performance under 1.2–1.7 V *vs.* RHE.

**Fig. 6:**
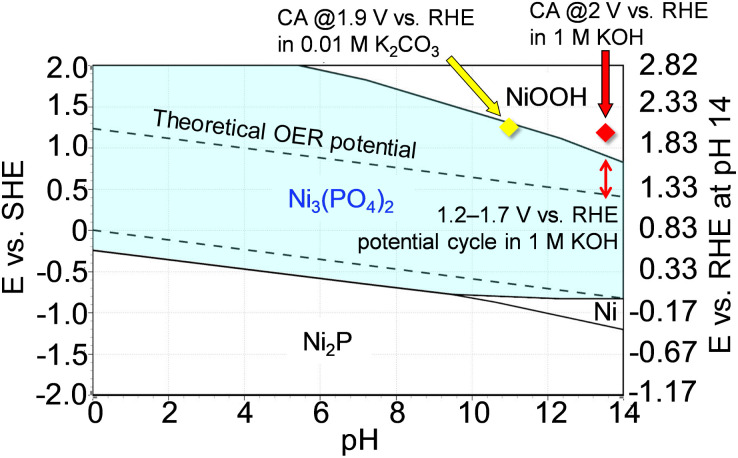
Pourbaix diagram of the Ni–P–H_2_O system at 298.15 K with the assumed concentration of soluble species and pressure set at 1.0 M and 1.0 bar, respectively. The scale on the right axis represents the RHE potential at pH 14. The converted potentials at pH 13.7 (1 M KOH) and 10.9 (0.01 M K_2_CO_3_) were calculated using the equation *E*_RHE_ = *E*_SHE_ + 0.0592 × pH.^66^

To support the robust stability of Ni_3_(PO_4_)_2_ within the abovementioned potential region, we performed additional theoretical simulations using HSC Chemistry software to generate Pourbaix diagrams. Pourbaix diagrams present the stable aqueous and solid species under certain conditions of potentials and pH values, with compounds highlighted in borders being the theoretically most stable species under those conditions. Fig. 6 shows the simulated Pourbaix diagram for the Ni–P–H_2_O system, revealing that Ni_3_(PO_4_)_2_ emerged as the most stable species under OER conditions in the potential cycle measurement, *i.e.*, up to ∼1.7 V *vs.* RHE at pH 13.7 (1 M KOH). This simulation further proved the stability of the Ni-based phosphates during OER measurements, indicating their role as active species for the OER. Moreover, to validate the dependence of Ni_3_(PO_4_)_2_ stability on electrode potential and pH of the electrolyte solution, long-term chronoamperometry tests were conducted at high potentials in both 1 M KOH and 0.01 M K_2_CO_3_ (pH 10.9),^67^ where NiOOH and Ni_3_(PO_4_)_2_ were identified as the most stable phases, respectively, as shown in Fig. 6. Fig. S15a† displays the chronoamperometric curve recorded at 2 V in 1 M KOH for 10 h, revealing a rapid decrease in current after applying the potential. Fig. S15b† exhibits STEM-EDX images of Ni_3_(PO_4_)_2_/Au after the chronoamperometry test indicating a substantial reduction in the composition of P throughout the sample. Additionally, the Raman spectrum of Ni_3_(PO_4_)_2_/Au shows the disappearance of characteristic peaks associated with Ni_3_(PO_4_)_2_, with the spectrum resembling that of Ni oxide,^68^ as shown in Fig. S15c.† These results show the dissolution of P from Ni_3_(PO_4_)_2_ and its electrochemical transformation into a Ni-based (oxyhydr)oxide, consistent with the Pourbaix diagram depicted in Fig. 6. Furthermore, Fig. S16† shows the results of a chronoamperometry test conducted for 10 h at 1.9 V *vs.* RHE using 0.01 M K_2_CO_3_, confirming the stability of Ni_3_(PO_4_)_2_ in a moderately alkaline electrolyte (pH 10.9) even at 1.9 V *vs.* RHE. These results align with the prediction of the Pourbaix diagram (Fig. 6), demonstrating the durability of Ni_3_(PO_4_)_2_ as an OER electrocatalyst across a wide range of conditions. If this electrocatalyst is used as the anode in AEMWEs, it is expected to deliver high electrolysis performance, and this performance can be maintained with a feed of diluted alkaline solution or pure water, owing to the thin membrane thickness and high OH^−^ conductivity of AEMs,^17,69^ which retain the Ni-based phosphates.

### Relationships among OER activity, atomic configurations and electronic orbitals

**Fig. 7:**
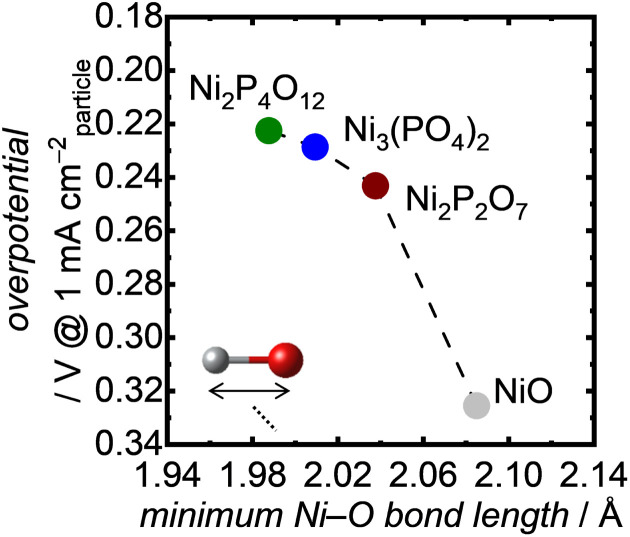
OER overpotentials at 1 mA cm_particle_^−2^ as a function of the minimum Ni–O bond length in the Ni-based phosphates and NiO.

To understand the excellent OER performance of Ni-based phosphates compared with NiO, we first examined the correlation between structural characteristics of the Ni-based electrocatalysts and their OER activities. It is well known that the atomic configurations of crystalline electrocatalysts strongly affect their OER activities.^43,70–73^Fig. 7 exhibits the OER overpotentials of both Ni-based phosphates and NiO plotted against the minimum Ni–O bond length in each crystal, demonstrating that short Ni–O bond lengths correspond to superior OER activity of the Ni-based OER electrocatalysts. Although various factors contribute to determining electrocatalytic activity, the Ni–O bond length can serve as a useful guideline for designing more efficient catalysts. This trend aligns well with our previous findings,^34,74^ which highlighted the influence of Fe–O bond length in Fe-based oxides on their OER activities, as shown in Fig. S17.† Additionally, the plots representing Ni-based compounds in Fig. S17† exhibit higher specific activities compared to those for Fe-based oxides, suggesting an enhancement in OER activity with Ni compared with Fe. This proves the merits of selecting Ni as an active metal to enhance OER activity.

**Fig. 8:**
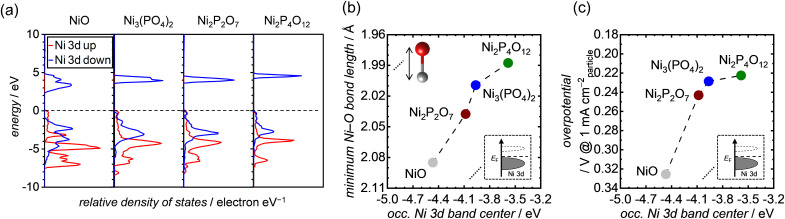
(a) DFT-calculated PDOS of the Ni 3d band for each specific Ni atom, which are the components of the shortest Ni–O bond length in each unit cell of NiO, Ni_3_(PO_4_)_2_, Ni_2_P_2_O_7_, and Ni_2_P_4_O_12_. The black dashed lines in the graphs represent the position of the Fermi energy level (*E*_F_). (b and c) The minimum Ni–O bond length and OER overpotential at 1 mA cm_particle_^−2^, respectively, as a function of the occupied Ni 3d band centers of each corresponding Ni atom, extracted from the PDOS in (a).

To gain deeper insights into the effect of the Ni–O bond length on OER activities, we performed DFT calculations and analyzed the bulk electronic orbitals of each Ni atom forming the minimum Ni–O bond in the Ni-based phosphates and NiO. The calculated PDOS of Ni 3d orbitals for both Ni-based phosphates and NiO are shown in Fig. 8a, with the occupied Ni 3d band centers extracted from the PDOS listed in Table S4.†Fig. 8b exhibits the minimum Ni–O bond length plotted against the energy levels of occupied Ni 3d band centers in the Ni-based phosphates and NiO, demonstrating a clear correlation. Fig. 8c displays the correlation between the OER overpotentials of the Ni-based phosphates and NiO and the energy levels of occupied Ni 3d band centers in these compounds, highlighting that a higher energy of occupied Ni 3d band centers results in superior OER overpotentials. Similar trends were previously reported by Lee *et al.*^75^ and Xiao *et al.*^76^ The favorable effect of the d bands on the OER can be explained through molecular orbital theory. In NiO_6_ coordination, Ni 3d and O 2p orbitals are hybridized to form Ni–O bonds, resulting in the splitting of orbitals into bonding and antibonding states. The strength of the bond between Ni and O relies on the extent of electron filling in these states. When the bonding states are filled with electrons, the bond becomes strong, while if the antibonding states are filled with electrons, the bond weakens. Bonding states are basically fully filled, whereas the filling of antibonding states depends on the energy levels of the Ni d-band center. The upshift of the d-band center leads to an increase in energy levels of antibonding states, resulting in less electron filling of the antibonding states, *i.e.*, the formation of strong bonds between active Ni sites on the catalyst surface and O* adsorbates.^76–79^ For example, Xiao *et al.*^76^ demonstrated that La^3+^-doping caused an upshift of the d-band center in NiO, strengthening its interaction with OER intermediates and thereby enhancing its OER activity. Furthermore, Tao *et al.*^79^ found that OER activities in p-type transition metal oxides, such as NiO, increased when the adsorption of O* on the surface became strong owing to the upshift of the d-band center and antibonding states of Ni.

**Fig. 9:**
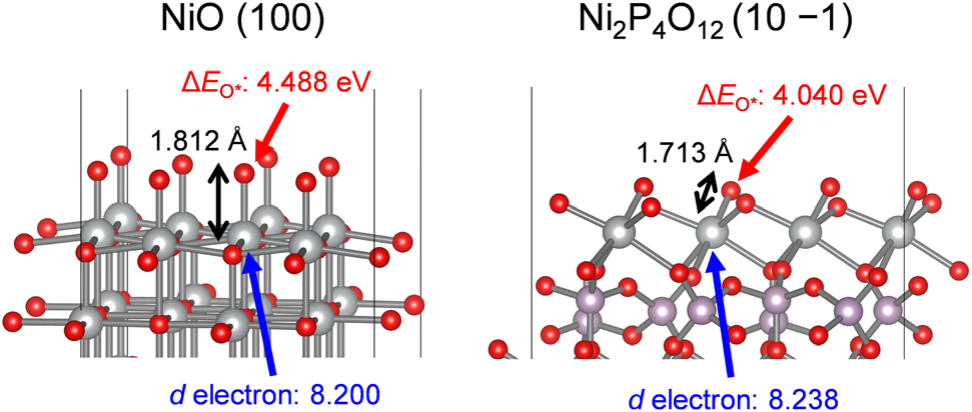
DFT-optimized surface slab models of NiO (100) and Ni_2_P_4_O_12_ (10 −1) showing the Ni–O* bond length, the number of d electrons in the Ni atoms, and Δ*E*_O*_. The gray, purple, and red spheres represent Ni, P, and O atoms, respectively.

To support the influence of the orbitals on the strength of O*adsorption, we performed DFT calculations using surface slab models of NiO and Ni_2_P_4_O_12_. The geometrically optimized bulk structures of NiO and Ni_2_P_4_O_12_ were cleaved to expose each Ni atom forming the minimum Ni–O bond, as depicted in Fig. S5b.† Optimized surface structures and adsorption energies of O* intermediates (Δ*E*_O*_) were then calculated and are summarized in Table 3 and Fig. 9. In bulk structures of NiO and Ni_2_P_4_O_12_, Ni_2_P_4_O_12_ possesses a shorter Ni–O bond length (1.989 Å) compared to NiO (2.098 Å), and a similar trend was observed for the DFT-optimized bond length between the surface Ni active site and the O* intermediate, namely, 1.713 Å and 1.812 Å for Ni_2_P_4_O_12_ and NiO, respectively. The variation in metal–O bond length between the bulk and surface is consistent with the literature.^43^ The trend of Ni–O bond length on surfaces indicates that adsorption of O* on the Ni_2_P_4_O_12_ surface is stronger than that on the NiO surface. Furthermore, the number of d electrons for the Ni atoms was obtained through DFT calculations, revealing that Ni on the surface of Ni_2_P_4_O_12_ possesses a greater number of d electrons than that on the surface of NiO, as shown in Table 3 and Fig. 9. The d electron occupancy in metals is known to affect the adsorption of O* and the subsequent OER activity, with O* adsorption being enhanced by the number of d electrons.^80^ Therefore, Ni on the surface of Ni_2_P_4_O_12_ with more d electrons leads to stronger O* adsorption than NiO due to their electronical configurations. Consequently, the calculated Δ*E*_O*_ values for NiO and Ni_2_P_4_O_12_ demonstrated that Ni_2_P_4_O_12_ can adsorb O* more strongly than NiO, resulting from the upshift of the d-band center and antibonding states of Ni. This finding aligns well with results depicted in Fig. 8c. Therefore, the enhanced OER activity of the Ni-based phosphates can be attributed to their more favorable electronic orbitals compared to NiO. The stronger O* adsorption and consequently higher OER activity observed in the phosphates compared to the oxide are consistent with findings on Co-based phosphates and CoO.^38^ This suggests that the catalytic advantage of metal phosphates over metal oxides can be extended to other transition metal phosphates.

**Table 3:** DFT-optimized bulk and surface slab models of NiO and Ni_2_P_4_O_12_ and the calculated number of d electrons in the Ni atoms and Δ*E*_O*_ for their surfaces

Catalyst	Bulk^a^	Surface slab^c^		
	Ni–O bond length^b^ [Å]	Ni–O* bond length^d^ [Å]	Number of d electrons in the Ni atom^e^ [—]	Δ*E*_O*_ [eV]
NiO	2.098	1.812	8.200	4.488
Ni_2_P_4_O_12_	1.989	1.713	8.238	4.040

a Geometrically optimized by DFT calculations.

b Minimum Ni–O bond length in DFT-optimized bulk structures.

c Simulated by DFT using surface slab models of (100) and (10 −1) facets for NiO and Ni_2_P_4_O_12_, respectively. To construct the slab models, the surfaces were cleaved to expose each Ni atom forming the minimum Ni–O bond in the DFT-optimized bulk structures of NiO and Ni_2_P_4_O_12_.

d Extracted bond lengths between the surface Ni atoms and adsorbed O* species.

e Obtained from DFT simulation of the surface Ni atoms with O* adsorbates.

**Fig. 10:**
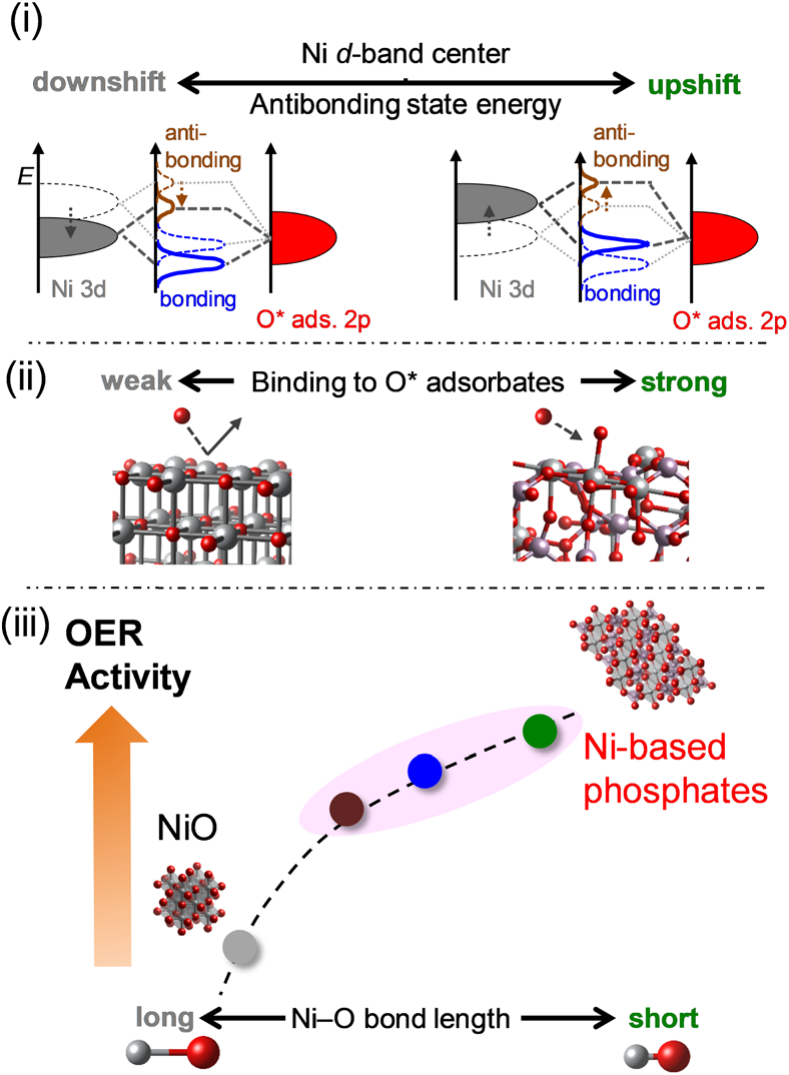
Schematic representation of the correlations between the OER activities of Ni-based electrocatalysts and their structural and electronic properties. (i) Effect of the upshift of the occupied Ni 3d band on the energy of antibonding states in the hybridized Ni–O bond. (ii) Impact of the energy of antibonding states on the binding strength of O* intermediates to Ni sites on catalyst surfaces. (iii) Reflection of Ni–O bond length, determining the subsequent OER activity.

The OER electrocatalysis of Ni-based phosphates and NiO concerning electronic influences is summarized in Fig. 10, according to the findings from this study. (i) Ni-based phosphates possess higher energy levels of antibonding states of the 3d–2p hybridized orbitals compared to NiO, according to the level of the d-band center for Ni active sites. (ii) The energy levels of antibonding states affect the bond strength between a Ni active site and an O* adsorbate in the OER process, with Ni-based phosphates showing stronger adsorption with OER intermediates than NiO. (iii) This bond strength is reflected in the Ni–O bond length in their crystal structures, which correlates with their OER activities, namely, shorter Ni–O bonds induced higher OER activity, and *vice versa*. Consequently, Ni-based phosphates exhibited better OER activity compared to NiO. Thus, the electronic structures of the Ni-based electrocatalysts intrinsically determine the OER activity, and the Ni–O bond length can be used as a design guideline for identifying efficient OER electrocatalysts, as this information is readily available from structural databases such as ICSD. Ni-based phosphates are promising OER catalysts for electrochemical water splitting, paving the way for the development of efficient green hydrogen production technology.

## Conclusions

We uncovered the outstanding electrocatalytic activities of Ni-based phosphates, including Ni_3_(PO_4_)_2_, Ni_2_P_2_O_7_, and Ni_2_P_4_O_12_, synthesized easily *via* the sol–gel method, for the anodic OER in electrochemical water splitting. These phosphates demonstrated superior OER activities compared to NiO, Ni_2_P, and IrO_2_ as well as previously reported state-of-the-art crystalline OER electrocatalysts. In addition, the Ni-based phosphates exhibited excellent durability during long-term potential cycle tests in the OER region, maintaining their crystal structure and elemental composition. Furthermore, we established a correlation between the OER activity and the Ni–O bond length in the crystal structures of the catalysts. DFT calculations revealed that the Ni-based phosphates possess more preferable electronic structures for the OER process than NiO, *i.e.*, the occupied Ni 3d orbitals in Ni-based phosphates are upshifted, strengthening the interaction with Ni active sites and O* intermediates, thereby enhancing their OER activities. Hence, Ni-based phosphates are promising OER electrocatalysts with excellent activity and durability. These findings offer new insights into the OER electrocatalysis of metal phosphates, and this study provides a rational design guideline for further improving Ni-based OER electrocatalysts and contributes to the innovation of electrochemical hydrogen production.

## Data availability

The experimental data supporting this article have been included in the article and the ESI.† The computational settings for DFT are described in the ESI.†

## Author contributions

T. Y., K. K. and Y. S. conceived this research project and designed the experiments. Y. N. and K. K. synthesized the catalysts. G. M. A. synthesized Ni_2_P. K. K. provided and characterized NiO. Y. S. and Y. N. carried out the structural characterization and electrochemical measurements of the catalysts. Y. S. carried out the computational calculations. All authors contributed to the writing of the manuscript.

## Conflicts of interest

There are no conflicts to declare.

## Supplementary Material

NA-OLF-D4NA00794H-s001
