# Supplementary material for: Oxygen evolution activity of nickel-based phosphates and effects of their electronic orbitals
Source: Nanoscale Adv. 2024 Dec 9;7(2):456–66. doi: 10.1039/d4na00794h (PMC11626464; doi:10.1039/d4na00794h)
Supplement: NA-007-D4NA00794H-s001 [file NA-007-D4NA00794H-s001.pdf]

Supplementary Information (SI) for Nanoscale Advances.
This journal is © The Royal Society of Chemistry 2024

Electronic Supplementary Information

Oxygen evolution activity of nickel-based phosphates and effects of their electronic orbitals

Yuuki Sugawara,*[,a] Yuto Nakase, [a] Gopinathan M Anilkumar, [ab,‡] Keigo Kamata,*[,c]
Takeo Yamaguchi*[,a]

[a]*Laboratory for Chemistry and Life Science, Institute of Integrated Research, Institute of Science Tokyo, R1-17, 4259 Nagatsuta-cho, Midori-ku, Yokohama, Kanagawa 226-8501, Japan*

[b]*R&D Centre, Noritake Co., Ltd., 300 Higashiyama, Miyochi-cho, Miyoshi 470-0293, Japan.*

[c]*Laboratory for Materials and Structures, Institute of Integrated Research, Institute of Science Tokyo, 4259 Nagatsuta-cho, Midori-ku, Yokohama, Kanagawa 226-8501, Japan*

*Corresponding author E-mail: yamag@res.titech.ac.jp; kamata.k.ac@m.titech.ac.jp;

sugawara.y.aa@m.titech.ac.jp.

Methods

Instruments

XRD analysis of the synthesized metal oxides was performed using a diffractometer (MiniFlex 600, Rigaku Corporation, Akishima, Japan; Cu Kα radiation, $\lambda = 1.5405$ Å, 40 kV, 15 mA) equipped with a high-speed one-dimensional detector (D/teX Ultra, Rigaku Corporation). Diffraction data were collected in the 2θ range of 10°–90° at 0.02° steps and a scan rate of 20° min^{-1}.

The specific surface areas of catalyst particles were determined through nitrogen sorption isotherms at 77 K recorded using a Micromeritics Tristar II automatic surface area and porosimetry analyzer (Micromeritics Instrument Corporation, Norcross, GA, USA), and the Brunauer–Emmett–Teller (BET) surface areas (S_{BET}) were calculated based on the relative pressure (P/P0) range of 0.05–0.30. Physisorbed water on the samples was dried by heating at 423 K for 2 h in vacuo prior to the measurements.

Scanning electron microscopy (SEM) images were obtained using a Hitachi S-5500 (Hitachi High-Technologies Co., Tokyo, Japan) at an acceleration voltage of 5.0 kV.

Transmission electron microscopy (TEM) images were obtained using a Hitachi H-8100 (Hitachi High-Technologies Co.) at 200 kV.

High-angle annular dark-field scanning TEM (HAADF-STEM) images were obtained using a Hitachi HD-2700 instrument (Hitachi High-Technologies Co.) with an accelerating voltage of 200 kV.

Energy-dispersive X-ray (EDX) spectroscopic elemental mappings were conducted using an EMAX Evolution system (HORIBA, Ltd., Kyoto, Japan).

Inductively coupled plasma–atomic emission spectroscopy (ICP–AES) analysis was performed using an ICPS-8100 spectrometer (Shimadzu Co., Kyoto, Japan).

Raman spectroscopy was conducted using a confocal Raman microscope (LabRAM HR Evolution, HORIBA Ltd.) equipped with a micro-Raman spectrometer (Renishaw In) in the backscattering geometry using a 532 nm excitation laser. The spectra were recorded with a resolution of 0.2 cm^{-1} using a 600 gr/mm grating at ambient temperature.

DFT calculations

In the DFT calculations, electronic exchange-correlation interactions were included using the generalized gradient approximation method with a Perdew–Burke–Ernzerhof functional (GGA-PBE).[1] For the on-site Coulombic interactions on the *d* electrons of Ni, the GGA + U method was applied with the Hubbard U parameter of 6.2 eV. The U value was chosen

according to the literature.[2, 3] Spin polarization was allowed in all calculations. An energy cutoff was set to 520 eV, and projector augmented wave pseudopotentials were used for all elements. Convergence thresholds for electronic state calculation and geometrical optimization were set to 1.0×10^{-6} eV and 2.0×10^{-2} eV Å^{-1} in energy and force, respectively. The Monkhorst–Pack scheme k-point grid sampling for the Brillouin zone was adjusted to maintain a k-point spacing of approximately ~0.025 Å^{-1} in each direction, as shown in Table S1, except for the surface calculation of $Ni_2P_4O_{12}$ (10 −1) and NiO (100) in which 4×3×1 and 4×4×1 k-points were used, respectively.

Supplementary Figures and Tables

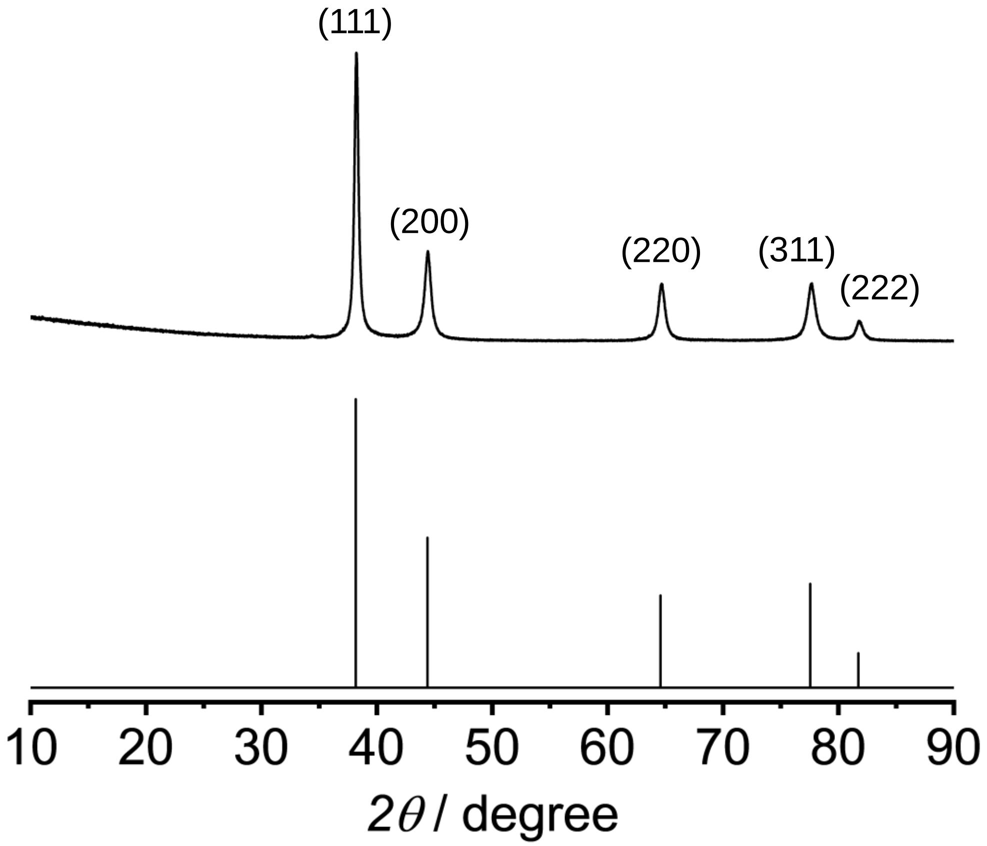

Fig. S1 Measured (upper) and theoretical (lower, ICSD: 64701) XRD patterns of Au.

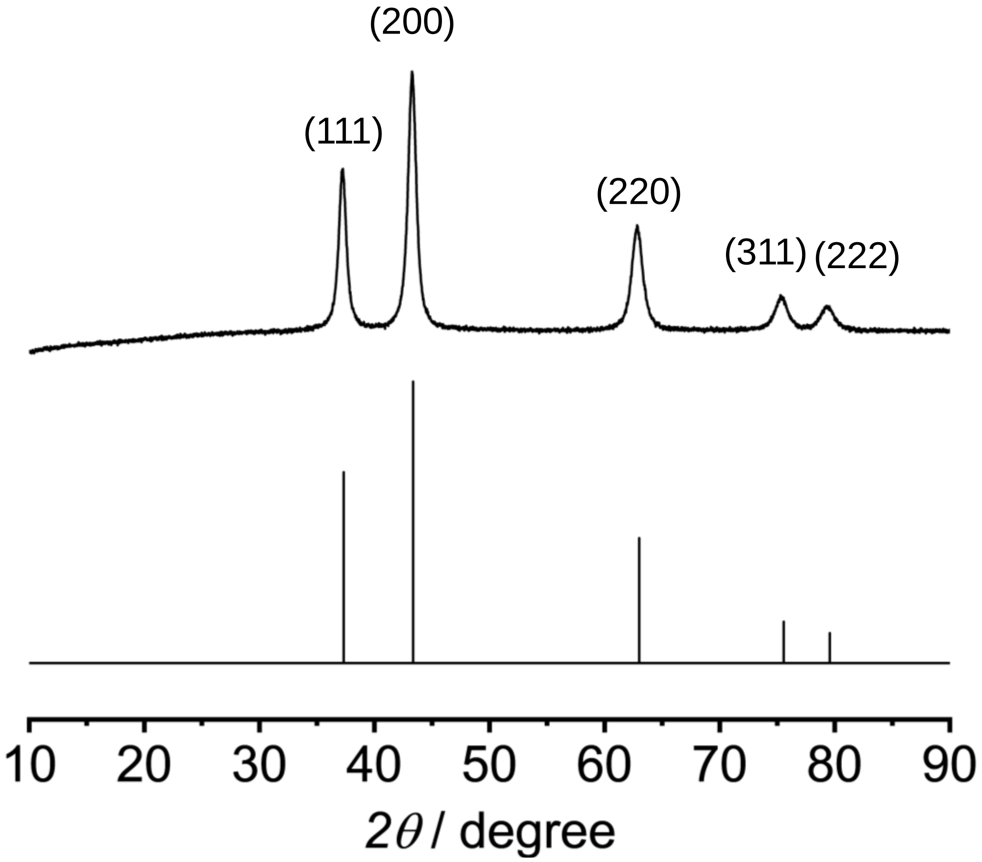

Fig. S2 Measured (upper) and theoretical (lower, ICSD: 28834) XRD patterns of NiO.

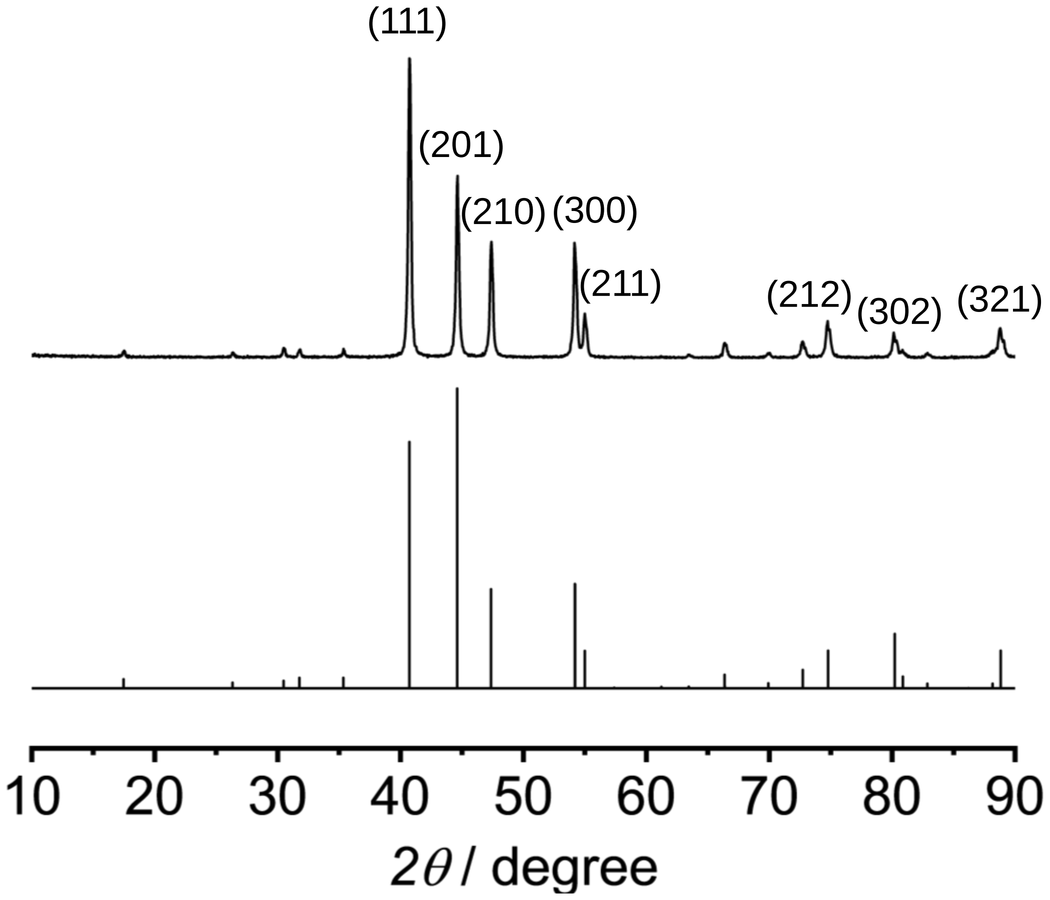

Fig. S3 Measured (upper) and theoretical (lower, ICSD: 27162) XRD patterns of Ni_2P.

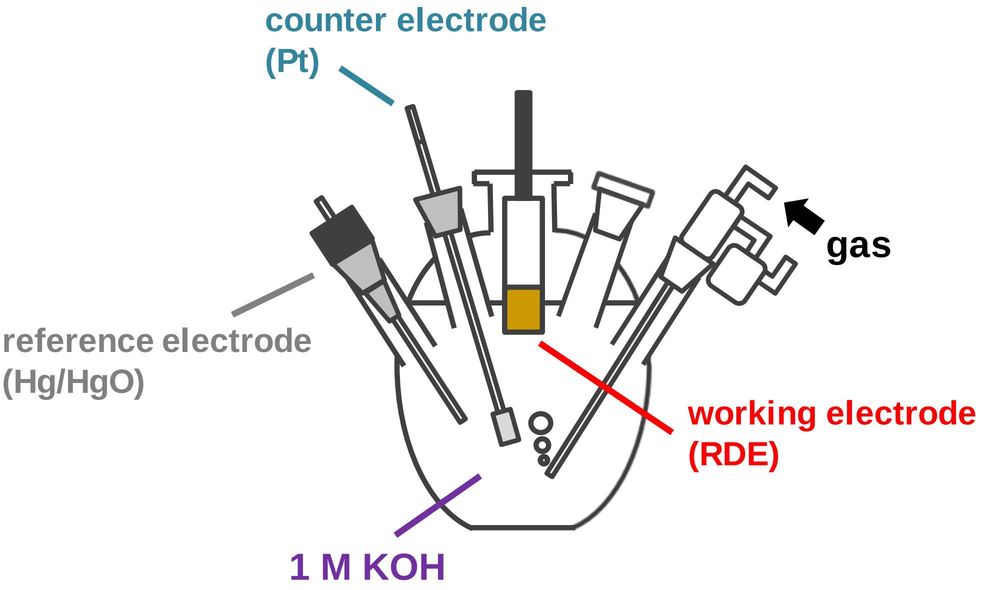

Fig. S4 Schematic representation of the setup for electrochemical OER measurements using RDE.

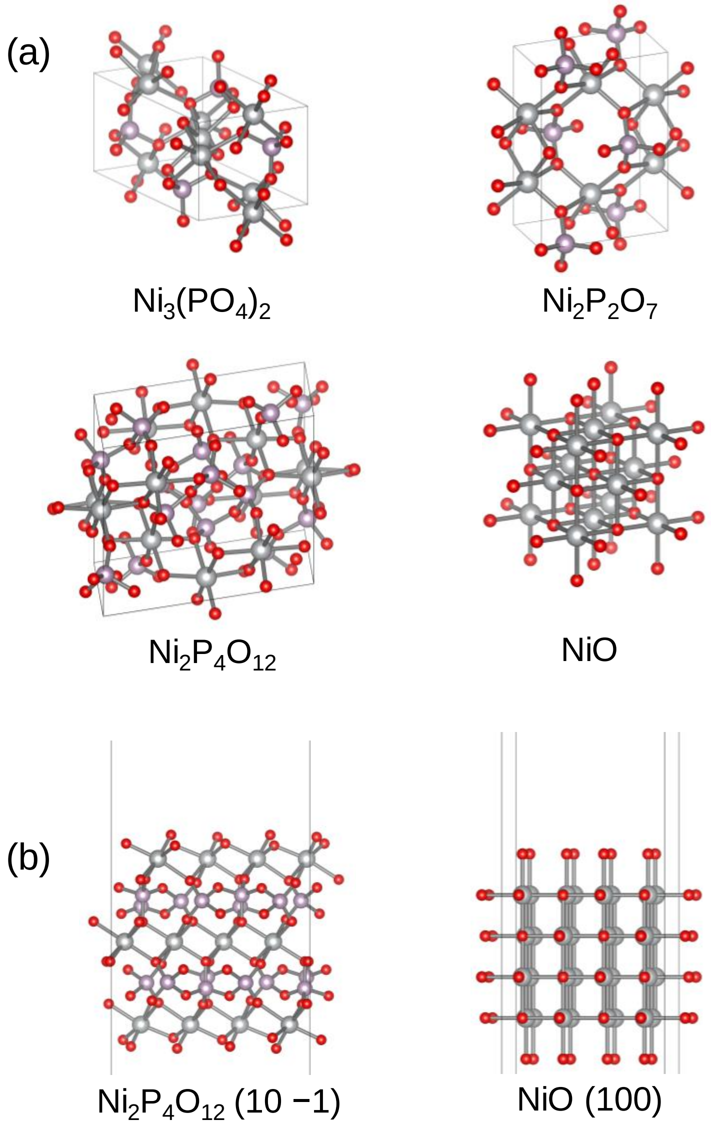

Fig. S5 (a) Unit cells and (b) surface slab models of the Ni-based phosphates and NiO used for DFT calculations, with O, Ni, and P atoms depicted as red, gray, and purple spheres, respectively.

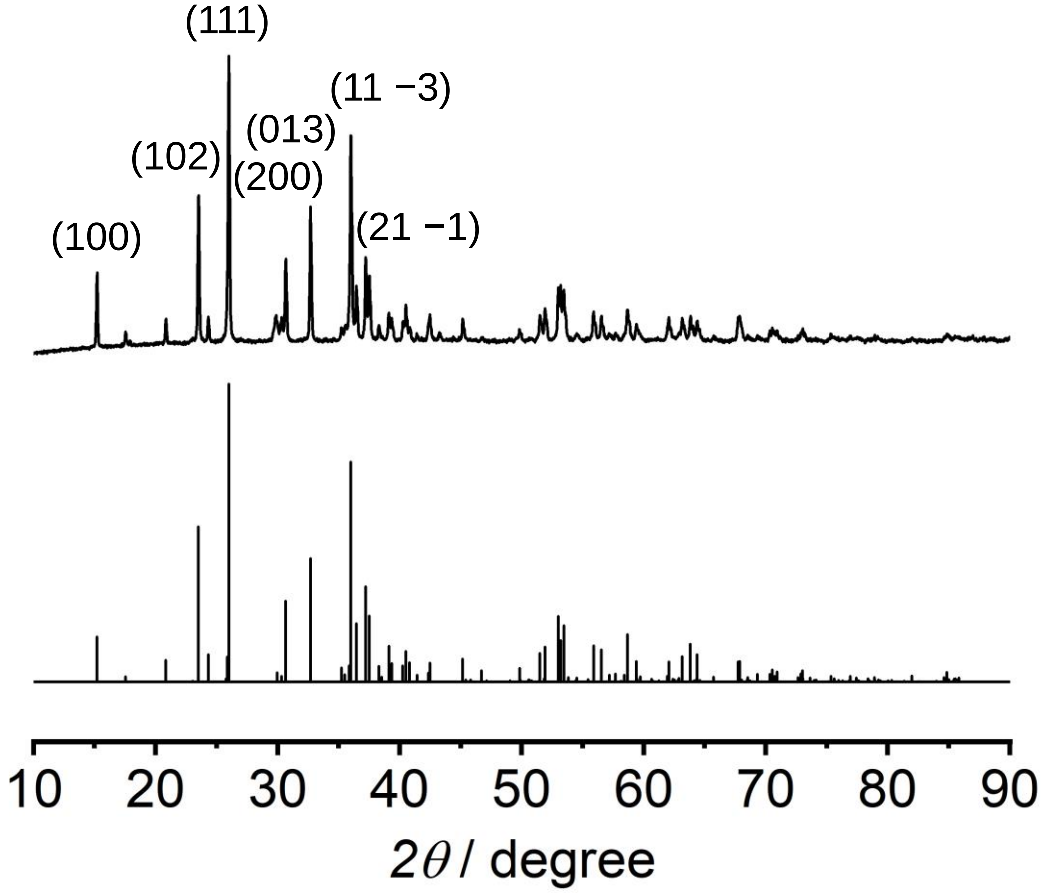

Fig. S6 Measured (upper) and theoretical (lower, ICSD: 153159) XRD patterns of $Ni_3(PO_4)_2$.

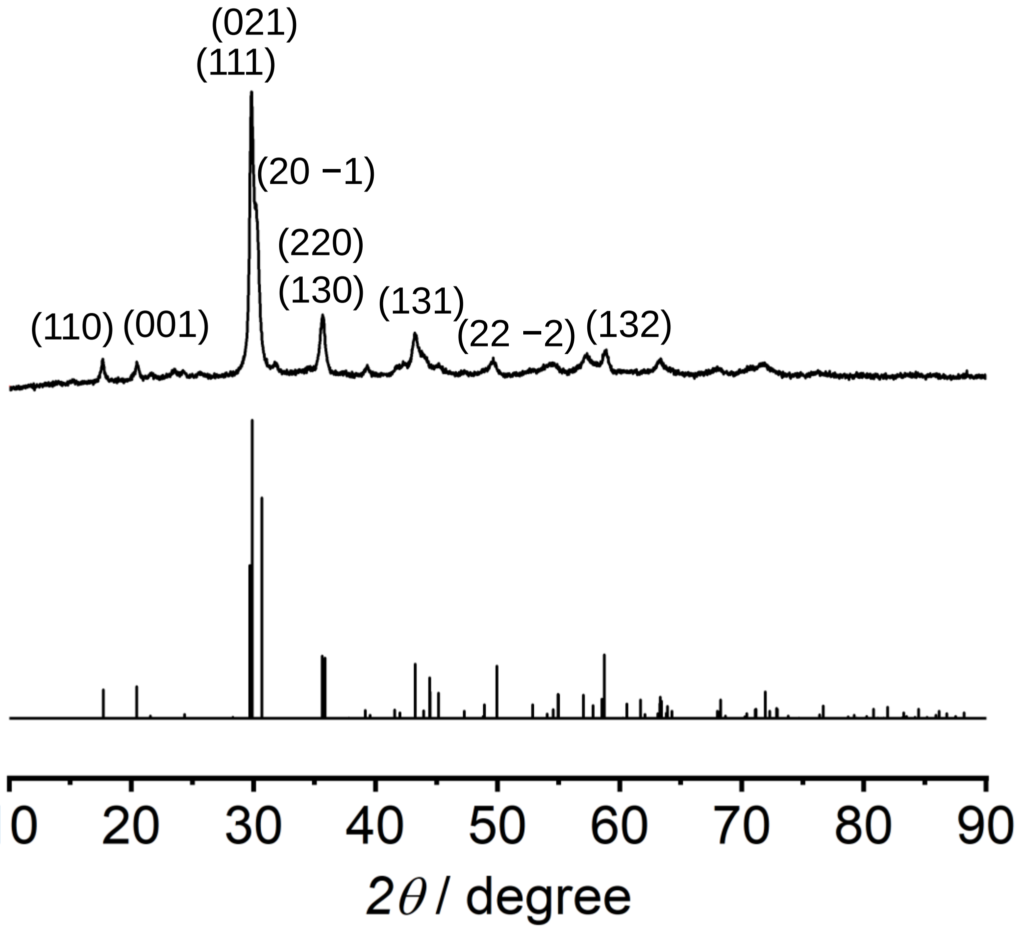

Fig. S7 Measured (upper) and theoretical (lower, ICSD: 30433) XRD patterns of $Ni_2P_2O_7$.

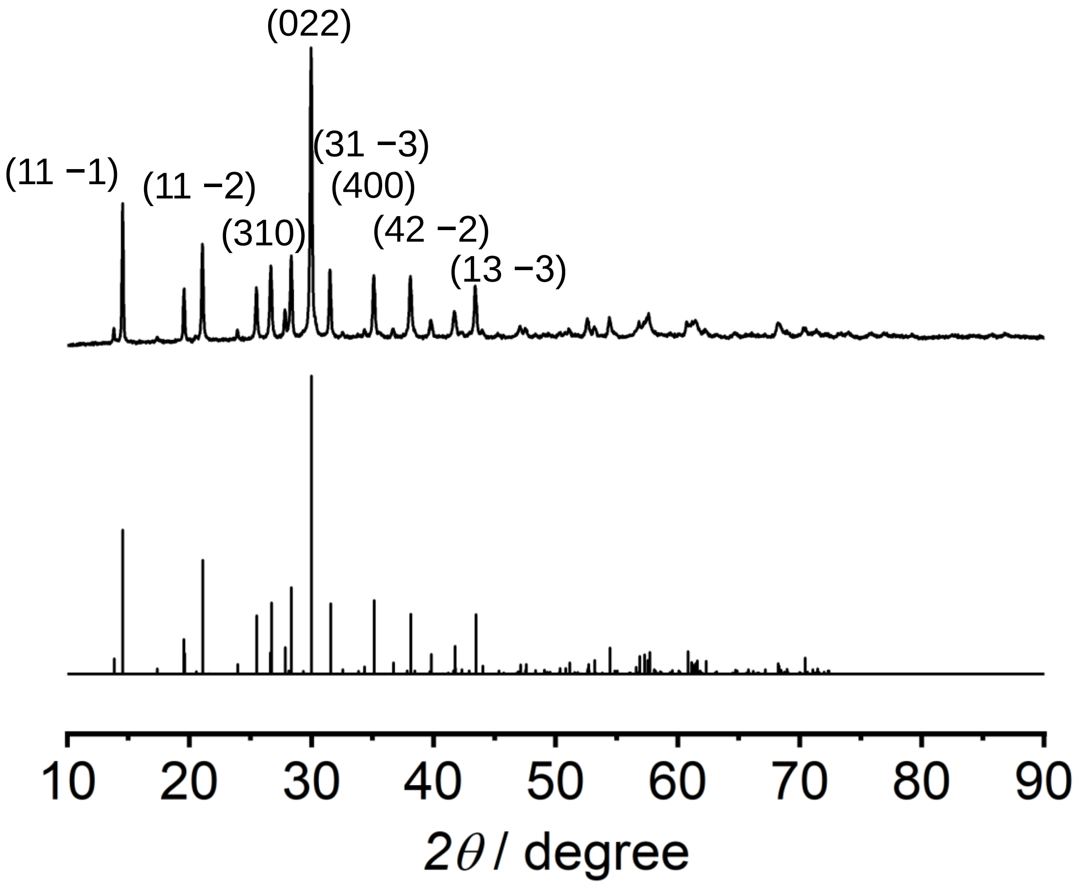

Fig. S8 Measured (upper) and theoretical (lower, ICSD: 409092) XRD patterns of $Ni_2P_4O_{12}$.

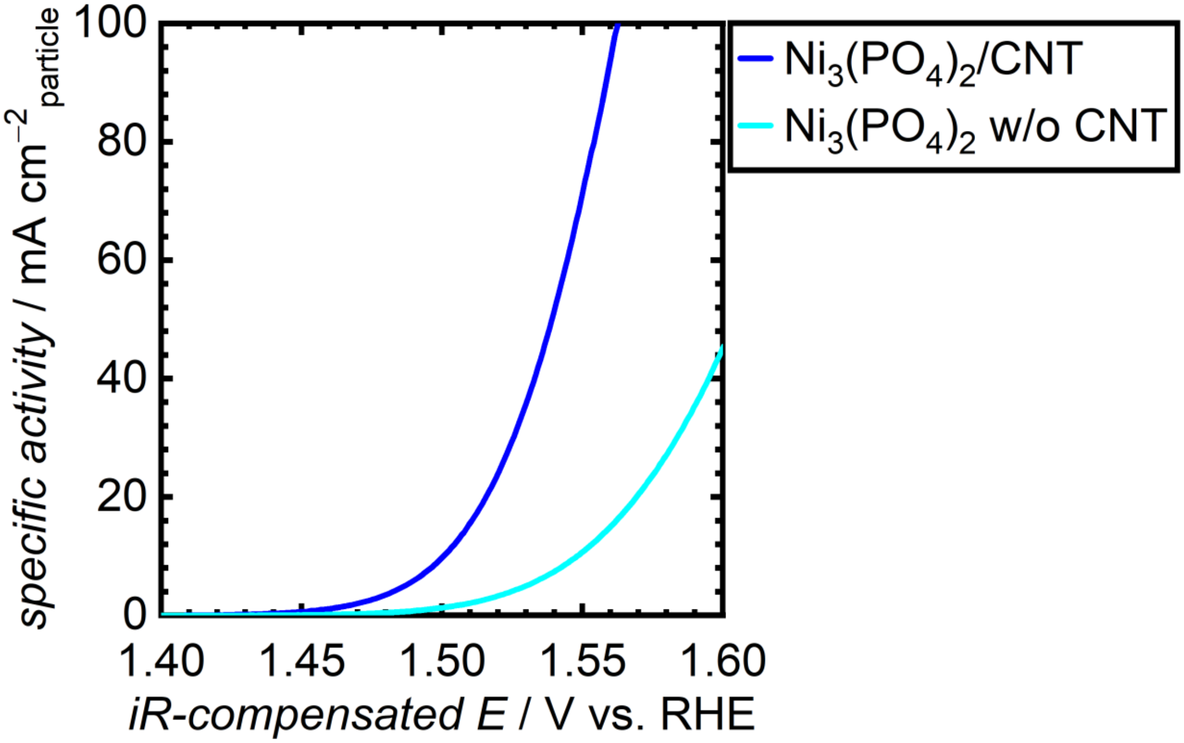

Fig. S9 Polarization curves showing OER-specific activity for the $Ni_3(PO_4)_2$/CNT and $Ni_3(PO_4)_2$ without CNT in 1 M KOH.

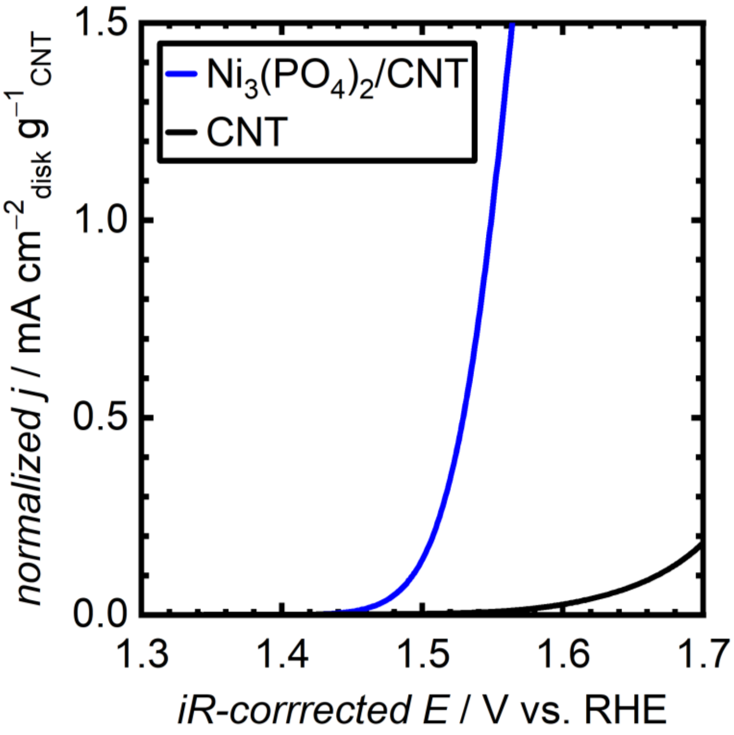

Fig. S10 Normalized polarization curves by CNT loading amount for the $Ni_3(PO_4)_2$/CNT and CNT in 1 M KOH.

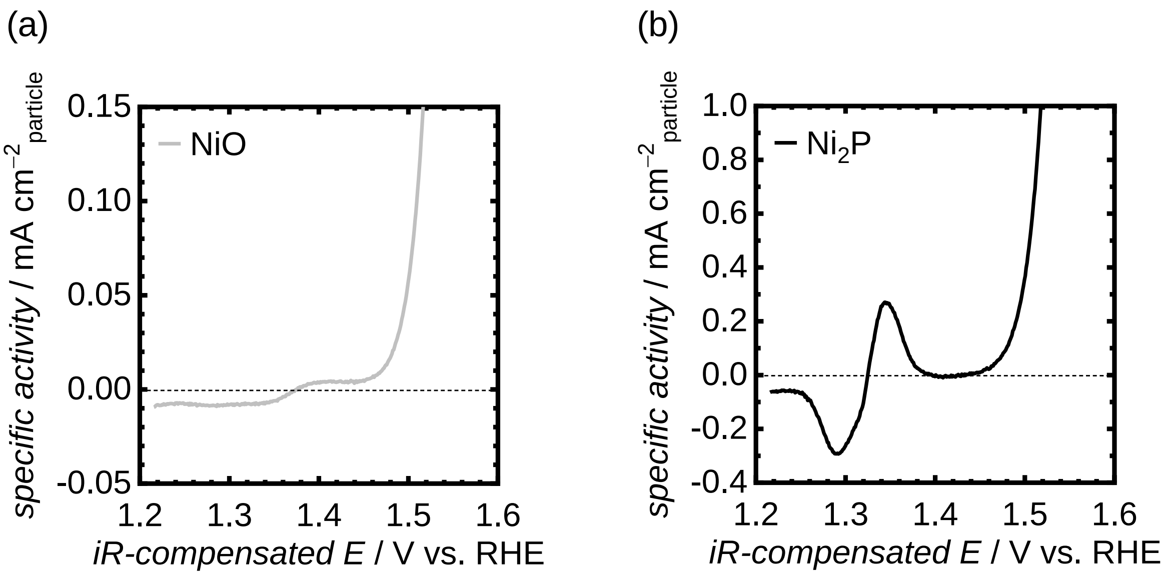

Fig. S11 Zoomed polarization curves for (a) NiO and (b) Ni2P in 1 M KOH.

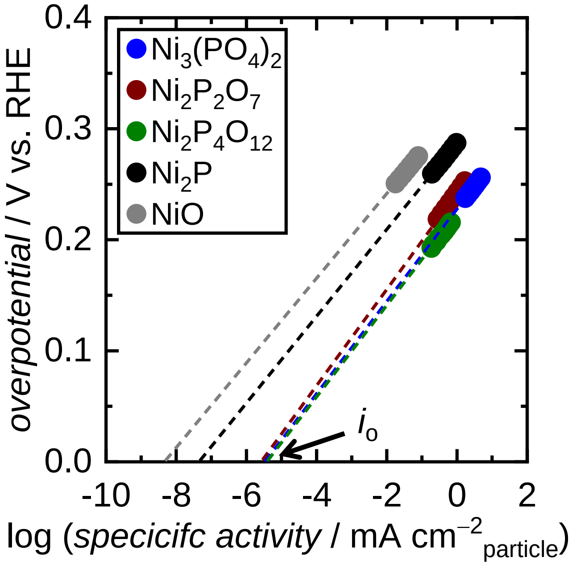

Fig. S12 Derivation of i_o from Tafel plots for the Ni-based phosphates, NiO, and Ni_2P

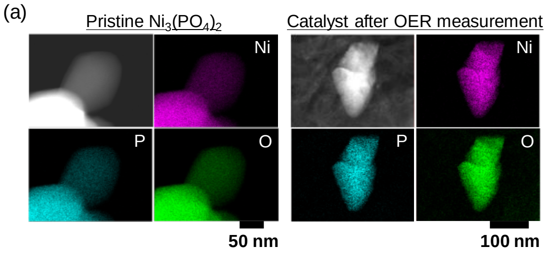

	Composition [wt%]		
	Ni	P	O
Theoretical	48	17	35
Initial	43	18	39
After OER	44	17	39

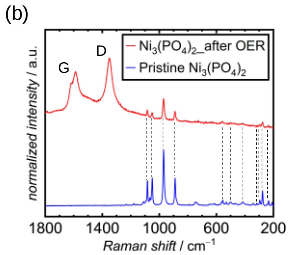

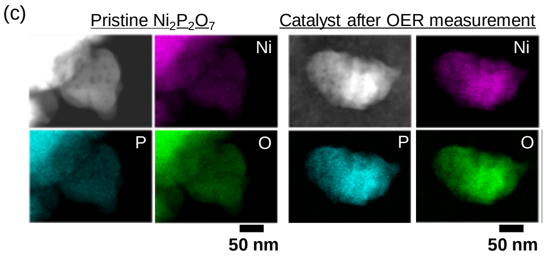

	Composition [wt%]		
	Ni	P	O
Theoretical	40	21	38
Initial	43	18	39
After OER	32	20	48

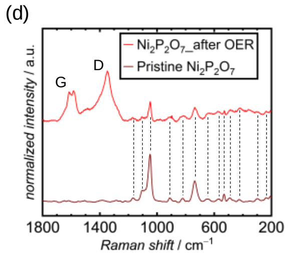

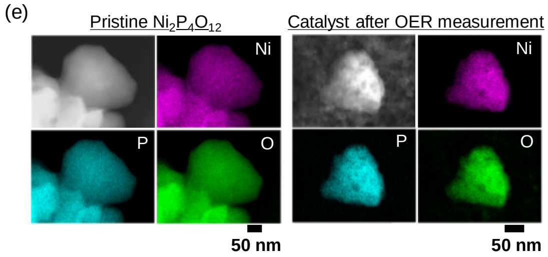

	Composition [wt%]		
	Ni	P	O
Theoretical	27	29	44
Initial	23	29	48
After OER	33	21	46

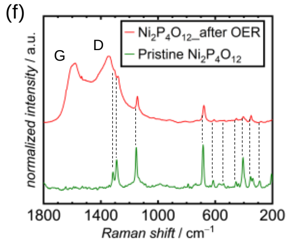

Fig. S13 (a, c, and e) STEM-EDX images and (b, d, and f) Raman spectra of the pristine and recovered catalysts after OER measurements for $Ni_3(PO_4)_2$, $Ni_2P_2O_7$, and $Ni_2P_4O_{12}$, respectively.

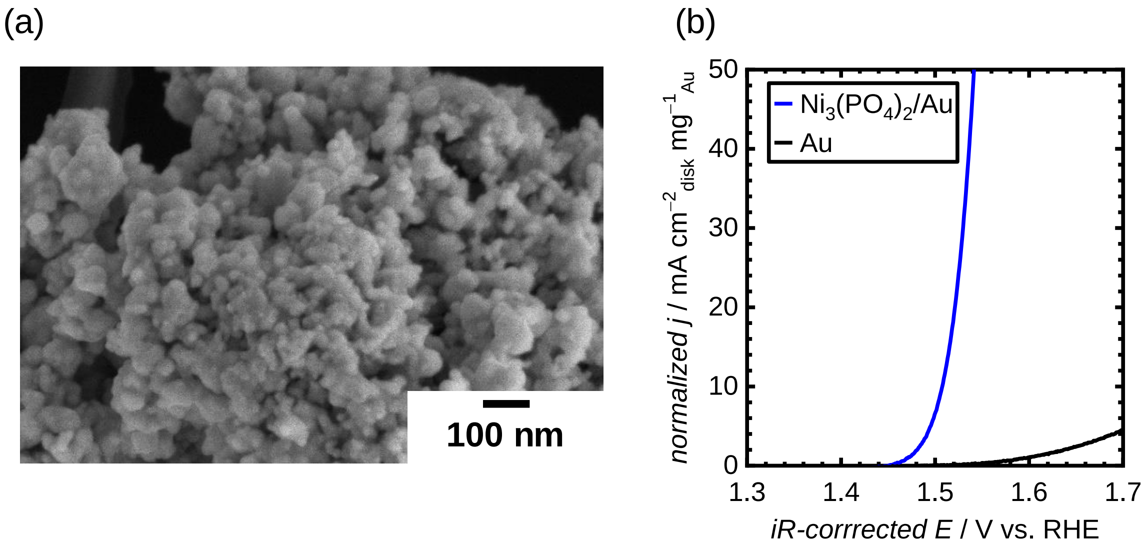

Fig. S14 (a) SEM image of the Au nanopowder. (b) Polarization curves in 1 M KOH for $Ni_3(PO_4)_2$/Au and Au without $Ni_3(PO_4)_2$.

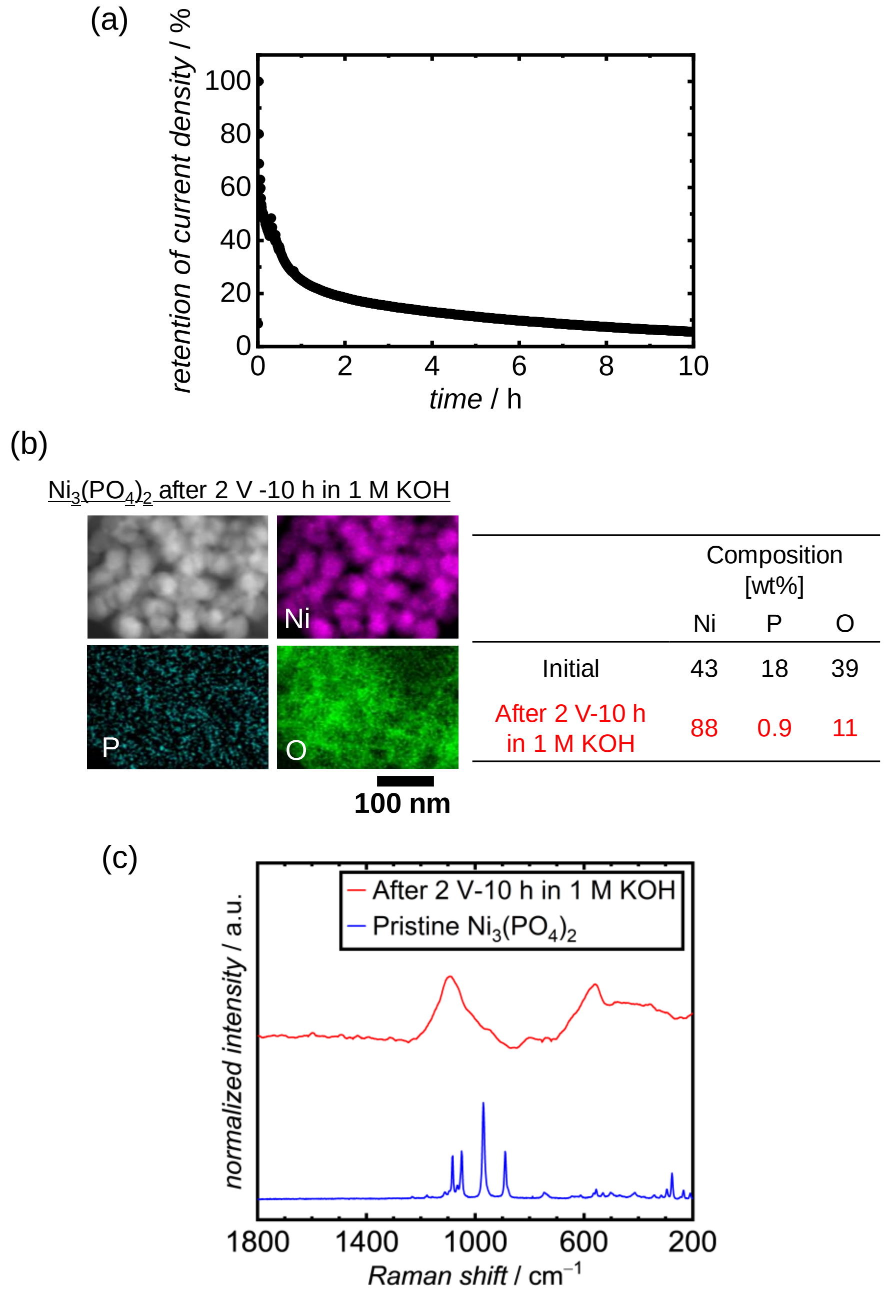

Fig. S15 (a) Chronoamperometric curve of $Ni_3(PO4)_2$/Au at 2 V vs. RHE in 1 M KOH. (b) STEM–EDX image and (c) Raman spectrum of $Ni_3(PO4)_2$/Au after 10 h at 2 V vs. RHE in 1 M KOH.

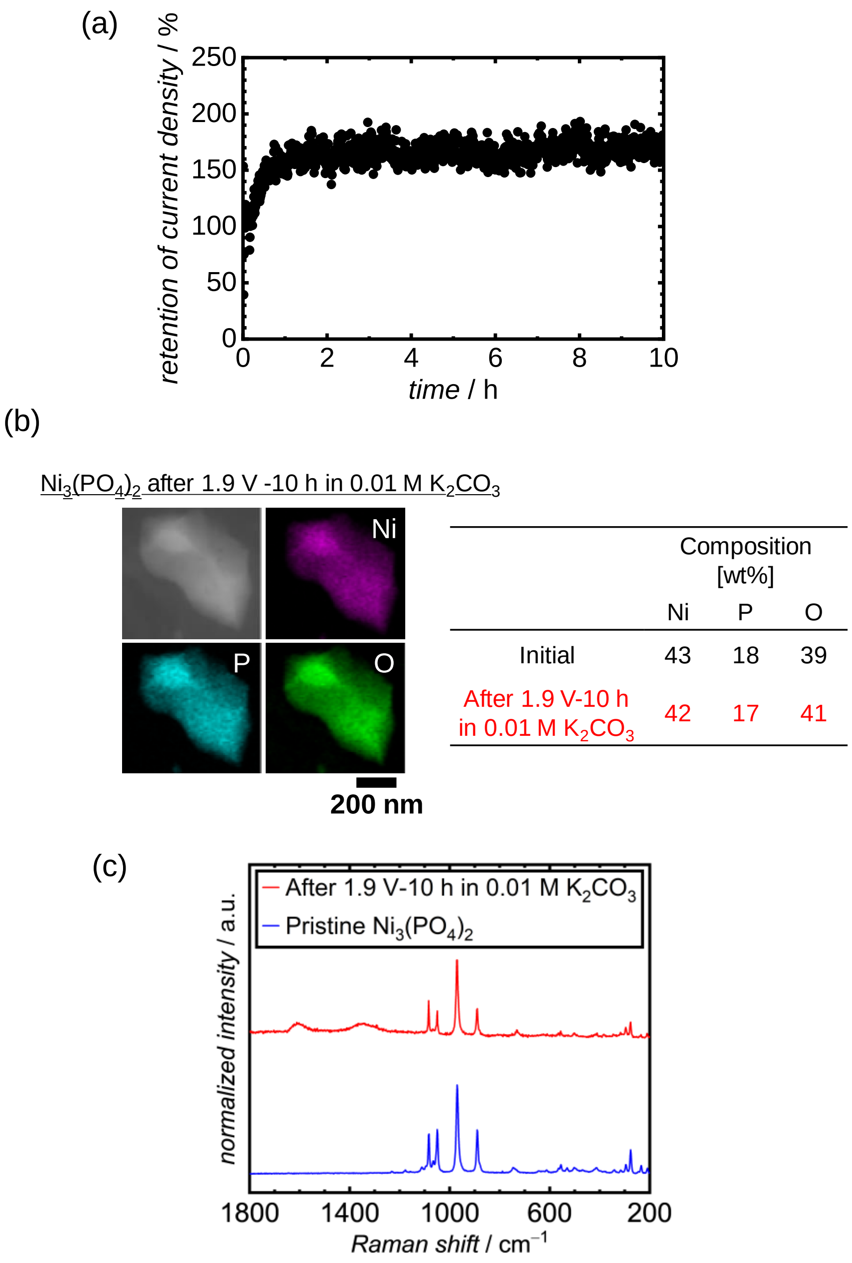

Fig. S16 (a) Chronoamperometric curve of $Ni_3(PO4)_2$/Au at 2 1.9 V vs. RHE in 0.01 M K_2CO_3. (b) STEM–EDX image and (c) Raman spectrum of $Ni_3(PO_4)_2$/Au after 1.9 V vs. RHE in 0.01 M K_2CO_3.

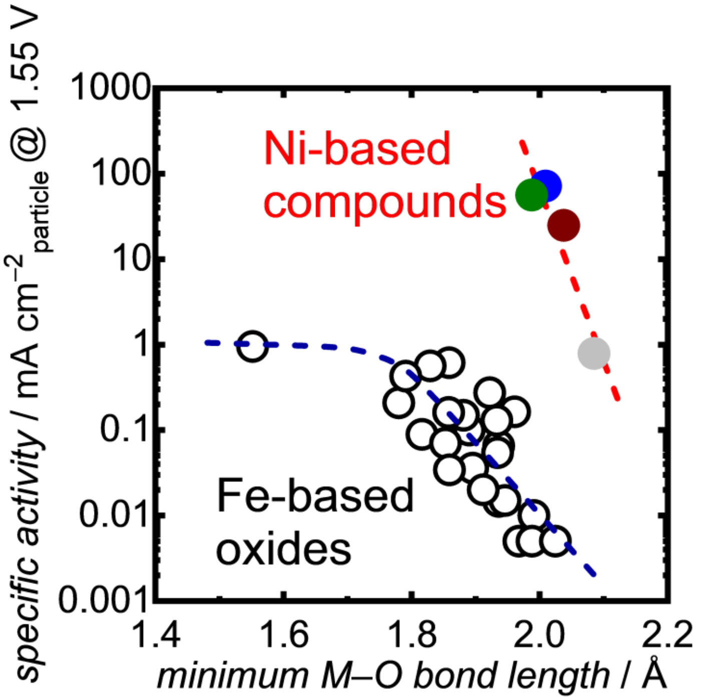

Fig. S17 Comparison of the trend between OER specific activities at 1.55 V as a function of the minimum M–O bond length for the Ni-based compounds and Fe-based oxides. The data for Fe-based oxides were adapted with permission from ref 4 Copyright 2022 John Wiley & Sons, Inc.

Table S1 Number of k-points and unit cell sizes of the calculated Ni-based phosphates and NiO.

Catalyst	Lattice vector length[a] [Å]			**k**-point
	a	b	c	
$Ni_3(PO_4)_2$	5.82400	4.69400	10.10100	7 × 8 × 4
$Ni_2P_2O_7$	6.50100	8.23900	4.48000	6 × 5 × 8
$Ni_2P_4O_{12}$	11.61100	8.21800	9.82600	3 × 5 × 4
NiO	4.17000	4.17000	4.17000	9 × 9 × 9

[a]Extracted from included structures in each ICSD code.

Table S2 Peaks observed in the Raman spectra of the Ni-based phosphates in Fig. 2c compared with literature values.

Catalyst	Raman shift [cm^{-1}]		Ref.
	Expt. in this study	Reported	
$Ni_3(PO_4)_2$	1049, 970, 890, 555, 412, 277, 233	1027 (ν_{PO3}), 948 ($\nu_{P–O–P}$), 917 ($\nu_{P–O–P}$), 563 (δ_{PO3}), 392 (δ_{PO3}, $\nu_{Ni–O}$), 283 ($\nu_{Ni–O}$, $\delta_{Ni–O}$), 245 ($\nu_{Ni–O}$, $\delta_{Ni–O}$)	5
$Ni_2P_2O_7$	1107, 1050, 912, 822, 736, 532, 490, 425	1300–1000 (ν_{PO3}), 990–920 ($\nu_{P–O–P}$), 870–670 ($\nu_{P–O–P}$), 600–350 (δ_{PO3})	6
$Ni_2P_4O_{12}$	1314, 1289, 1153, 685, 614, 572, 539, 455, 437, 405, 354, 338, 288	1318 (ν_{PO3}), 1289 (ν_{PO3}), 1154 (ν_{PO3}), 686 (δ_{PO3}), 618 (δ_{PO3}), 577 (δ_{PO3}), 542 (δ_{PO3}), 456 (δ_{PO3}), 441 (δ_{PO3}), 407 ($\delta_{P–O–P}$), 353 ($\delta_{P–O–P}$), 338 ($\delta_{P–O–P}$), 291 ($\nu_{Ni–O}$, $\delta_{Ni–O}$)	7

Table S3 Comparison of OER-specific activity in 1 M KOH with the other state-of-the-art nonprecious metal-based crystalline catalysts and IrO_2.

Catalyst	Specific activity @1.55 V [mA $cm^{-2}{}_{particle}$]	Ref
$Ni_2P_4O_{12}$ (current study)	56	–
Contracted microcrystal NiFe-LDH	4.6	8
microcrystal NiFe-LDH	2.5	8
IrO_2	2.0	9
Ni_2P-F	1.7	10
$BaNiO_3$	1.4	11
Ni_5P_4	1.3	12
BSCF	1.1	13
NiFe-ODS-LDH	1.1	14
$La_{1.6}Sr_{0.4}NiO_4$	1.0	15
$CaFe_2O_4$	1.0	16
$LiCo_{0.8}Fe_{0.2}O_2$	0.97	17
$La_{1.67}Sr_{0.33}NiO_{3.8}$	0.80	15
Ni_5P_2	0.80	12

Table S4 DFT-calculated occupied Ni 3*d* band centers for the Ni-based phosphates and NiO, which were extracted from Fig. 8a.

Catalyst	Occupied Ni 3*d* band center [eV]
$Ni_3(PO_4)_2$	−3.96
$Ni_2P_2O_7$	−4.08
$Ni_2P_4O_{12}$	−3.58
NiO	−4.47

References

1. J. P. Perdew, K. Burke and M. Ernzerhof, *Phys. Rev. Lett.*, 1996, **77**, 3865–3868.
2. R. P. Forslund, W. G. Hardin, X. Rong, A. M. Abakumov, D. Filimonov, C. T. Alexander, J. T. Mefford, H. Iyer, A. M. Kolpak, K. P. Johnston and K. J. Stevenson, *Nat. Commun.*, 2018, **9**, 3150.
3. X. P. Wang, L. B. Li, M. Z. Shi, Y. Q. Wang, G. D. Xu, K. Yuan, P. P. Zhu, M. N. Ding and Y. W. Chen, *Chem. Sci.*, 2022, **13**, 11639–11647.
4. Y. Sugawara, S. Ueno, K. Kamata and T. Yamaguchi, *ChemElectroChem*, 2022, **9**, e202101679.
5. A. Agarwal, S. Majumder and B. R. Sankapal, *J. Energy Storage*, 2023, **58**, 106396.
6. R. Chen, Q. L. Gao, Y. Q. Qiao, J. Guo and E. J. Liang, *Scr. Mater.*, 2022, **214**, 114653.
7. K. Viswanathan, V. U. Nayar and G. Aruldhas, *J. Chem. Sci.*, 1985, **95**, 463–469.
8. Z. C. Zheng, D. Wu, G. Chen, N. Zhang, H. Wan, X. H. Liu and R. Z. Ma, *Carbon Energy*, 2022, **4**, 901–913.
9. Y. Sugawara, K. Kamata, A. Matsuda and T. Yamaguchi, *ACS Appl. Energy Mater.*, 2023, **6**, 7928–7934.
10. S. D. Ghadge, O. I. Velikokhatnyi, M. K. Datta, K. Damodaran, P. M. Shanthi and P.

N. Kumta, *J. Electrochem. Soc.*, 2021, **168**, 064512.

11. M. Retuerto, F. Calle-Vallejo, L. Pascual, P. Ferrer, A. Garcia, J. Torrero, D. Gianolio, J. L. G. Fierro, M. A. Pena, J. A. Alonso and S. Rojas, *J. Power Sources*, 2018, **404**, 56–63.

12. P. Zhang, Y. R. Lu, C. S. Hsu, H. G. Xue, T. S. Chan, N. T. Suen and H. M. Chen, *Chem. Commun.*, 2020, **56**, 8071–8074.

13. J. Suntivich, K. J. May, H. A. Gasteiger, J. B. Goodenough and Y. Shao-Horn, *Science*, 2011, **334**, 1383–1385.

14. J. A. Carrasco, R. Sanchis-Gual, A. Seijas-Da Silva, G. Abellán and E. Coronado, *Chem. Mater.*, 2019, **31**, 6798–6807.

15. R. Sankannavar, K. C. Sandeep, S. Kamath, A. K. Suresh and A. Sarkar, *J. Electrochem. Soc.*, 2018, **165**, J3236–J3245.

16. Y. Sugawara, K. Kamata, A. Ishikawa, Y. Tateyama and T. Yamaguchi, *ACS Appl. Energy Mater.*, 2021, **4**, 3057–3066.

17. Y. L. Zhu, W. Zhou, Y. B. Chen, J. Yu, M. L. Liu and Z. P. Shao, *Adv. Mater.*, 2015, **27**, 7150–7155.
